# LncRNA Hnf4αos exacerbates liver ischemia/reperfusion injury in mice via Hnf4αos/Hnf4α duplex-mediated PGC1α suppression

**DOI:** 10.1016/j.redox.2022.102498

**Published:** 2022-10-06

**Authors:** Chaoqun Wang, Hongjun Yu, Shounan Lu, Shanjia Ke, Yanan Xu, Zhigang Feng, Baolin Qian, Miaoyu Bai, Bing Yin, Xinglong Li, Yongliang Hua, Liqian Dong, Yao Li, Bao Zhang, Zhongyu Li, Dong Chen, Bangliang Chen, Yongzhi Zhou, Shangha Pan, Yao Fu, Hongchi Jiang, Dawei Wang, Yong Ma

**Affiliations:** a Department of Minimal Invasive Hepatic Surgery, The First Affiliated Hospital of Harbin Medical University, Harbin, China; b Key Laboratory of Hepatosplenic Surgery, Ministry of Education, China; c Department of Hepatic Surgery, The First Affiliated Hospital of Harbin Medical University, Harbin, China; d The First Department of General Surgery, The Affiliated Hospital of Inner Mongolia Minzu University, Tongliao, China; e Department of Pediatric Surgery, The First Affiliated Hospital of Harbin Medical University, Harbin, China; f Department of Intensive Care Unit, The First Affiliated Hospital of Harbin Medical University, Harbin, China; g Department of Ultrasound, The First Affiliated Hospital of Harbin Medical University, Harbin, China; h Department of Anorectal Surgery, The First Affiliated Hospital of Harbin Medical University, Harbin, China

**Keywords:** *Hnf4αos*, Liver, Ischemia/reperfusion, PGC1α, Reactive oxygen species

## Abstract

LncRNAs are involved in the pathophysiologic processes of multiple diseases, but little is known about their functions in hepatic ischemia/reperfusion injury (HIRI). As a novel lncRNA, the pathogenetic significance of hepatic nuclear factor 4 alpha, opposite strand (*Hnf4αos*) in hepatic I/R injury remains unclear. Here, differentially expressed *Hnf4αos* and *Hnf4α* antisense RNA 1 (*Hnf4α-as1*) were identified in liver tissues from mouse ischemia/reperfusion models and patients who underwent liver resection surgery. *Hnf4αos* deficiency in *Hnf4αos*-KO mice led to improved liver function, alleviated the inflammatory response and reduced cell death. Mechanistically, we found a regulatory role of *Hnf4αos*-KO in ROS metabolism through PGC1α upregulation. *Hnf4αos* also promoted the stability of *Hnf4α* mRNA through an RNA/RNA duplex, leading to the transcriptional activation of miR-23a and miR-23a depletion was required for PGC1α function in hepatoprotective effects on HIRI. Together, our findings reveal that *Hnf4αos* elevation in HIRI leads to severe liver damage via Hnf4αos/Hnf4α/miR-23a axis-mediated PGC1α inhibition.

## Highlights

• High expression of *Hnf4αos* was associated with a severe damage in I/R mouse model.
• *Hnf4αos* exacerbates I/R injury by reducing ROS clearance.
• *Hnf4αos* promotes *Hnf4α* mRNA stability through a RNA-RNA duplex.
• *Hnf4αos* involved in I/R process via *Hnf4αos/Hnf4α/miR-23a*-mediated PGC1α inhibition.

## Graphical abstract

**Figure:**
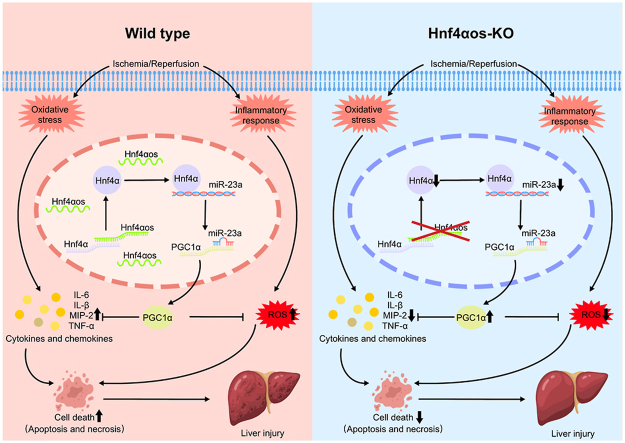

## Abbreviations

lncRNAs: long noncoding RNAs
Hnf4αos: hepatic nuclear factor 4 alpha, opposite strand
Hnf4α: hepatocyte nuclear factor 4 alpha
PGC1α: PPARγ coactivator-1α
ROS: reactive oxygen species; I/R, ischemia/reperfusion
A/R: anoxia/reoxygenation
ALT: alanine aminotransferase
AST: aspartate aminotransferase
ELISA: enzyme-linked immunosorbent assay
TUNEL: terminal deoxynucleotidyl transferase-mediated dUTP nick-end labeling
MDA: malondialdehyde
4-HNE: 4-hydroxynonenal
SOD: superoxide dismutase
CAT: catalase
GPX: glutathione peroxidase
LDH: lactate dehydrogenase

## 1 Introduction

Hepatic ischemia/reperfusion injury (HIRI) is a common pathological process that occurs in several clinical scenarios, such as complex liver resection, liver transplantation, and hemorrhagic shock. During this process, the initial ischemic injury causes direct hepatocyte damage, and subsequent blood flow reflux further aggravates liver dysfunction and injury due to the propagation of reactive oxygen species (ROS), macrophage activation and inflammatory cytokines, which trigger cell death [1,2]. However, the underlying molecular mechanisms of ischemia/reperfusion (I/R) injury remain largely unknown.

Long noncoding RNAs (lncRNAs) are defined as single-stranded RNA molecules spanning more than 200 nucleotides that are involved in multilevel gene expression regulation, including epigenetic modification, and transcriptional and posttranscriptional progression [3]. According to the proximity to protein coding genes in the genome, lncRNAs are generally placed into five categories: sense, antisense, bidirectional, intronic, and intergenic lncRNAs [4]. Currently, several studies have highlighted the significant roles of lncRNAs in the pathogenesis of liver disease. For instance, lncRNA *HULC* is upregulated in hepatocellular carcinoma and enhances hepatocarcinogenesis by promoting the phosphorylation of YB-1 via the ERK pathway [5]; lncRNA *ANRIL* alleviates liver fibrosis and hepatic stellate cell (HSC) activation via the AMPK pathway [6]; and lncRNA *CCAT1* promotes nonalcoholic fatty liver disease (NAFLD) by increasing LXRα transcription [7]. Nevertheless, in the case of hepatic I/R injury, little is known about lncRNAs in hepatic I/R injury. Thus, a deeper understanding of the molecular mechanisms underlying the pathogenic process of hepatic I/R is required to uncover potential lncRNA-targets for developing promising therapeutic strategies.

Furthermore, we have identified a novel lncRNA hepatic nuclear factor 4 alpha, opposite strand (*Hnf4αos*), a natural antisense transcript (NAT) of hepatocyte nuclear factor 4 alpha (Hnf4α), which was aberrantly upregulated in mouse I/R models. Although *Hnf4αos* has been reported, little information is available for regarding its molecular function [8,9]. PPARγ coactivator 1 alpha (PGC1α) is well known as a metabolic regulator in the physiological process of oxidative phosphorylation (OXPHOS), the tricarboxylic acid (TCA) cycle and ROS metabolism [[10], [11], [12]]. Intriguingly, our previous studies have demonstrated that PGC1α is an important regulator of ROS metabolism that reduces cell death, ameliorates the sterile inflammatory response and alleviates oxidative stress-induced liver damage during hepatic I/R insult [13]. Moreover, several lines of evidence, including data from bioinformatic analysis and determination of oxidative stress levels, suggest a close link between the lncRNA *Hnf4αos* and PGC1α. Thus, we further investigated the effects of *Hnf4αos* on I/R progression and the underlying mechanisms between *Hnf4αos* and PGC1α.

## 2 Material and methods

### 2.1 Human liver samples

Human liver samples were obtained from subjects who underwent partial hepatectomy due to hepatic hemangioma. All procedures involving human samples were approved by the Ethics Committee of the First Affiliated Hospital of Harbin Medical University and patient informed consent was obtained. We listed the detailed clinical information of the hemangioma patients in Supplementary Table S3.

### 2.2 Animals

Male C57BL/6 mice, hepatocyte-specific *Hnf4αos* knockout (*Hnf4αos*-KO) mice and wild-type (WT) mice (8 weeks old) were housed in specific pathogen-free (SPF) conditions and raised following institutional guidelines for animal care. *Hnf4αos*-KO mice were obtained by CRISPR/Cas9 methods as described previously [14]. *Hnf4αos*-KO mice were generated by crossing *Hnf4αos*-floxed mice with Albumin-Cre mice (Jackson Laboratory. Bar Harbor, ME, USA) on the C57BL background. The donor vector containing the fourth exon of the *Hnf4αos* gene was floxed by two loxP sites. All animal experiments were performed in accordance with the standard protocols of the Committee on the Use of Live Animals in Teaching and Research of Harbin Medical University, Harbin, China.

### 2.3 Mouse hepatic I/R injury model

The procedures for partial hepatic ischemia have been described previously [15]. Mice were housed in a specific pathogen-free and temperature-controlled environment with a 12-h light/dark cycle. Briefly, the mice were anesthetized with pentobarbital sodium (50 mg/kg), and a midline laparotomy was performed. An atraumatic clip was placed across the left lateral and median lobes of the liver (∼70%). After 75 min of partial hepatic ischemia, the clip was removed for initial reperfusion. Sham control mice underwent the same operation without vascular clamping.

### 2.4 Cell A/R treatment model

Cellular anoxic conditions were established and maintained in a modular incubator chamber (Biospherix, Lacona, NY, USA) by continuous gas flow with a 1% O_2_, 5% CO_2_ and 94% N_2_ gas mixture. After incubation under hypoxia for 6 h, the cells were incubated under normoxic conditions with 95% air and 5% CO_2_ for the indicated times (0, 3, 6, 12, 24h). The medium and cells were collected for further analysis.

### 2.5 Cell culture and treatment

Mouse hepatocytes were isolated by a modified in situ collagenase perfusion technique as previously described [15]. Hepatocyte purity and viability typically exceeded 99 and 95%, respectively. Primary hepatocytes and L02 cell lines (Type Culture Collection of the Chinese Academy of Science) were cultured in DMEM supplemented with 10% fetal bovine serum and 1% penicillin-streptomycin in a 5% CO_2_/water-saturated incubator at 37 °C.

### 2.6 Immunofluorescence assay

Paraffin-embedded tissue sections were used for immunofluorescence as described previously [16]. The liver sections were incubated with primary antibody against Ly6G (Cell Signaling Technology) (1:500) (31469), and the slides were incubated with corresponding fluorescence-labeled secondary antibody (ThermoFisher) (1; 1000) (A32744) for further staining.

### 2.7 ROS detection

Cellular reactive oxygen species (ROS) levels were estimated as previously described [17]. For intracellular ROS levels, cells were incubated in medium containing 10 μM dihydroethidium (DHE) (Invitrogen, USA) for 30 min at 37 °C in the dark. The medium was switched to fresh medium before fluorescence detection. The relative ROS levels, which are proportional to the fluorescence intensity, were quantified using Image-Pro Plus software.

### 2.8 Luciferase reporter assay

We predicted potential Hnf4α binding sites on the PGC1α and miR-23a promoters using the JASPAR database, and the PGC1α 3’-untranslated region (UTR) contains conserved miR-23a binding sites as reported previously [18]. We then cloned the candidate binding sites in an SV40 driven luciferase reporter plasmid. Briefly, luciferase activity was assessed using a luciferase assay kit (Promega, Madison, WI, USA). HEK-293T cells containing specific plasmids and 1 ng pRL-TK Renilla luciferase plasmid were seeded into 24-well plates. After 48 h, we used the dual luciferase reporter assay system (Promega) to measure luciferase activity according to the manufacturer's instructions.

### 2.9 Ribonuclease protection assay (RPA)

A ribonuclease protection assay (RPA) and quantitative RT-PCR were performed to detect the RNA-RNA duplex. Total RNA from primary hepatocytes was isolated as described previously [19]. The RNA samples were treated with DNAse Ⅰ (Sigma, 12.5 units/ml) and RNase A (QIAgen, 200 ng/ml) to remove residual DNA and single-stranded RNAs. Finally, the solutions were incubated for 40 min at 37 °C for further qRT-PCR.

### 2.10 Electrophoretic mobility shift assay (EMSA)

An electrophoretic mobility shift assay (EMSA) was performed as described previously [12]. The oligonucleotides used in EMSA were as follows: Hnf4α/miR-23a wt, 5’-GATCAGCTGGCCCCTGAAAACCTTGTTTAAC-3’ and 3’-CTAGTCGACCGGGGACTTTTGGAACAAATTG-5’. Hnf4α/miR-23a mut, 5’-GATCAGCTCCCCCCTAAAAAACTTGTTTAAC-3’ and 3’-CTAGTCGAGGGGGGATTTTTTGAACAAATTG-5’.

### 2.11 Statistical analysis

All data are expressed as the mean ± SD. Significant differences between groups were determined by ANOVA, with Bonferroni correction for continuous variables and multiple groups. Two-tailed Student's *t*-test was used for comparison of a normally distributed continuous variable between 2 groups. The level of significance was set at a p value less than 0.05 for all analyses.

Further details of the experimental materials and procedures are described in the Supplementary Files.

## 3 Results

### 3.1 LncRNA *Hnf4αos* is elevated during hepatic I/R injury

**Fig. 1:**
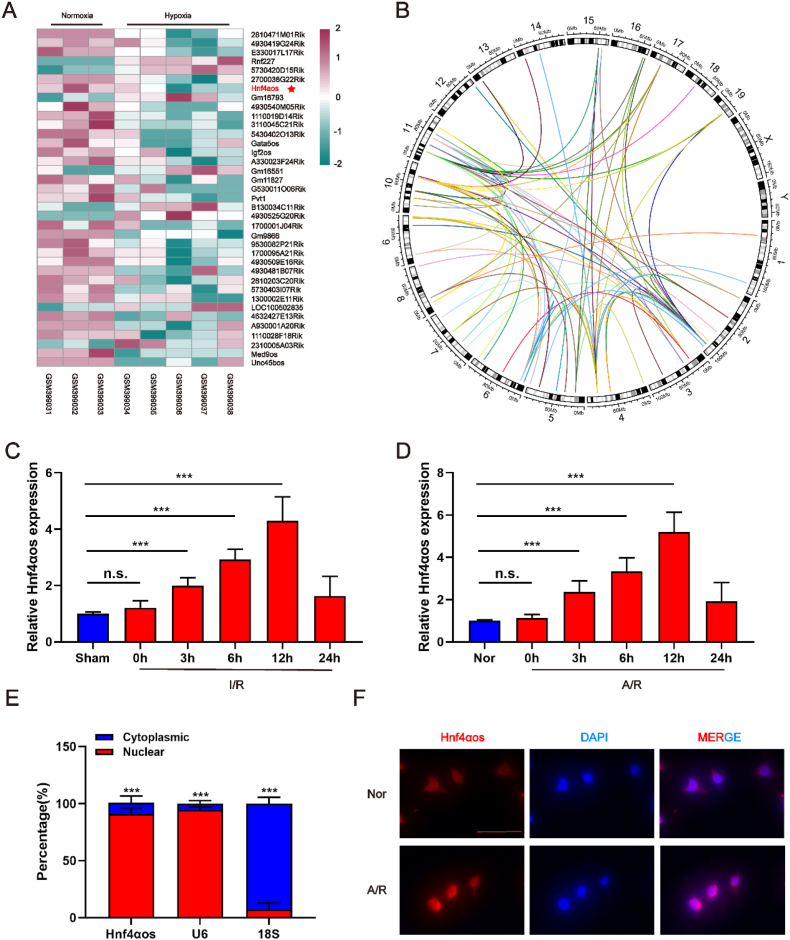
LncRNA *Hnf4αos* is elevated during hepatic I/R injury. **(A)** Heatmaps generated using the RNA expression of members detected by the DEG analysis. The expression of RNAs was visualized in color saturation; the expression level of genes was indicated by the colors (3 mice in the normoxia group and 5 mice in hypoxia group). **(B)** Genomic distance between lncRNAs and correlated with the oxidative stress, inflammatory response and apoptosis genes in KEGG. (The outer ring shows the distribution of the chromosomes of the mouse; The internal lines indicate that the top lncRNA-mRNA pairs) **(C)***Hnf4αos* expression was assessed by qRT-PCR in mouse liver I/R models. **(D)***Hnf4αos* expression was assessed by qRT-PCR in primary hepatocytes after A/R treatment. **(E)** Levels of cytoplasmic and nuclear *Hnf4αos* in primary hepatocytes. **(F)** The cellular locations and expression changes of *Hnf4αos* were analyzed by RNA-FISH. The scale bar represents 50 μm. n.s. P > 0.05, *P < 0.05, **P < 0.01, ***P < 0.001. (For interpretation of the references to color in this figure legend, the reader is referred to the Web version of this article.)

Several lncRNAs were differentially expressed in the GEO data-set (GSE15891) with exposure to chronic anoxia and our heatmap demonstrated the marked differentially expressed lncRNAs related to oxidative stress, inflammatory response and apoptosis pathways (Fig. 1A). For examining the relationships of lncRNAs and traget genes, the top-ranked lncRNAs and mRNAs correlated oxidative stress/inflammatory response/apoptosis resident on different chromosomes (Fig. 1B). Among the top-ranked differentially expressed lncRNAs, only *Hnf4αos* was enriched in adult mouse liver tissue (Supplementary Table S1, 2). Thus, *Hnf4αos* was selected for further investigation during hepatic I/R injury. To explore the role of lncRNA *Hnf4αos* in HIRI, we first detected the expression levels of *Hnf4αos* in murine hepatic I/R and hepatocyte A/R models, and *Hnf4αos* was found to be increased after reperfusion. The human-derived lncRNA, *Hnf4α-as1*, was also found to be differentially expressed in clinical liver samples from patients who underwent partial hepatectomy (Fig. 1C–D, Supplementary Fig. S1). Furthermore, cellular fractionation of hepatocytes followed by qRT-PCR implied that *Hnf4αos* was predominantly expressed in the nuclei of hepatocytes rather than other compartments, compared with U6 (localized in the nucleus) and 18S (localized in the cytoplasm) expression (Fig. 1E). Moreover, a fluorescence in situ hybridization (FISH) assay was performed to detect the locations of and changes in *Hnf4αos* in mouse hepatocytes after A/R treatment. The results showed that the fluorescence intensity of *Hnf4αos* was markedly enriched in hepatocyte nuclei and significantly elevated in the A/R group compared with the normoxic group (Fig. 1F). Therefore, we identified *Hnf4αos* as a novel therapeutic target in the pathogenic process of hepatic I/R injury.

### 3.2 *Hnf4αos* exacerbates liver damage induced by hepatic I/R insult

**Fig. 2:**
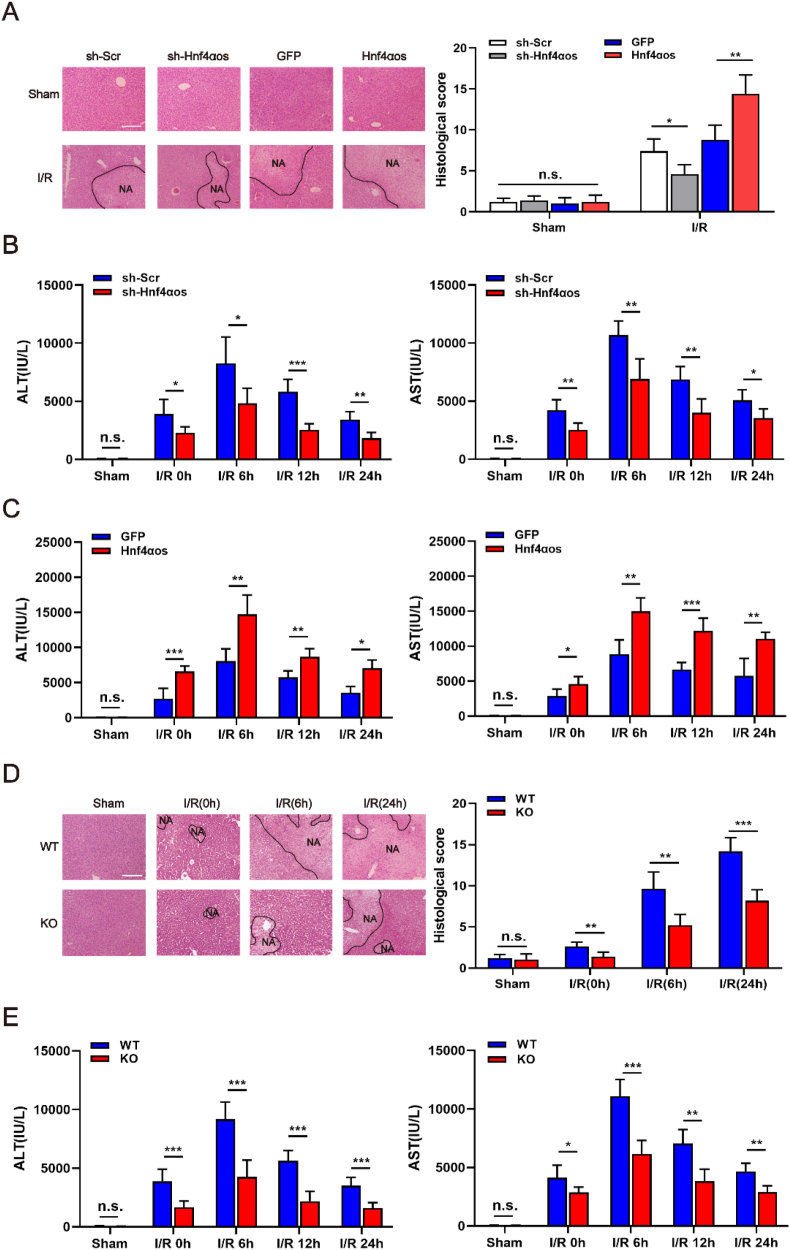
*Hnf4αos* deteriorates liver damage induced by hepatic I/R insult. **(A)** Images (100 × magnification) of H&E-stained liver sections and representative histopathological scores after the transfection of adenovirus vectors. The scale bar represents 200 μm. **(B–C)** Serum levels of aminotransferases (ALT and AST) were detected in the mice subjected to I/R after the transfection of adenovirus vectors. **(D)** Images of H&E-stained liver sections and representative histopathological scores in *Hnf4αos*-KO and WT mice. The scale bar represents 200 μm. **(E)** Serum levels of aminotransferases (ALT and AST) were detected in the *Hnf4αos*-KO and WT mice subjected to I/R operation. n.s. P > 0.05, *P < 0.05, **P < 0.01, ***P < 0.001.

To evaluate the potential effects of *Hnf4αos* on liver damage after hepatic I/R in mice, we altered the expression level of endogenous *Hnf4αos* by tail vein injection with *Hnf4αos* overexpression and downregulation adenoviral vectors (Supplementary Fig. S2A). When we knocked down *Hnf4αos* expression in mice, no statistical significance in sham mice was found, and I/R induced tissue necrosis was markedly ameliorated in the liver by silencing *Hnf4αos* expression, whereas, *Hnf4αos* overexpression worsened pathological changes (hemorrhagic change, inflammatory cell infiltration and focal necrosis) in I/R liver tissue (Fig. 2A). Additionally, serum aminotransferase (ALT and AST) levels were also significantly decreased in *Hnf4αos* knockdown mice, and ectopic expression of *Hnf4αos* exhibited the opposite effect compared with control mice (Fig. 2B and C). Thus, we concluded that *Hnf4αos* exacerbated liver damage induced by HIRI insult.

To obtain more evidence supporting the role of *Hnf4αos* in I/R-induced liver injury, we generated *Hnf4αos*-knockout (*Hnf4αos*-KO) and *Hnf4αos*-wild-type (*Hnf4αos*-10.13039/100010269WT) mice (Supplementary Figs. S2B and C). Subsequently, *Hnf4αos*-KO mice were subjected to a 75-min I/R operation. As expected, histological H&E staining showed considerable amelioration of tissue necrosis levels by *Hnf4αos* knockout (Fig. 2D). Moreover, *Hnf4αos*-KO mice exhibited reduced release of ALT and AST in serum compared with *Hnf4αos*-WT mice (Fig. 2E). Of note, serum aminotransferases were significantly lower in the low *Hnf4α-as1* group, suggesting less liver injury and better liver function after partial hepatectomy (Supplementary Fig. S2D). Overall, these observations suggest that *Hnf4αos* inhibition plays a protective role in hepatic I/R injury.

### 3.3 *Hnf4αos* knockout inhibits the inflammatory response during hepatic I/R injury

**Fig. 3:**
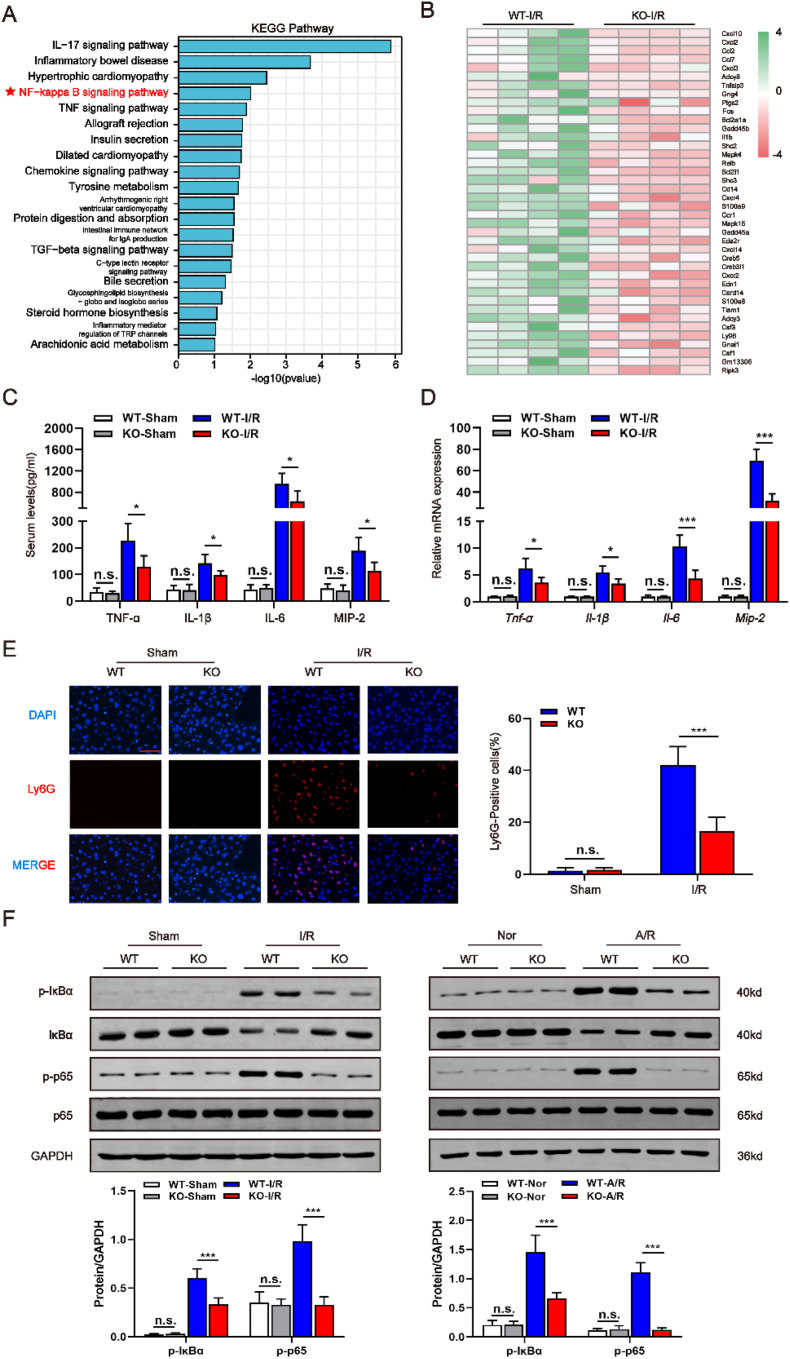
Hnf4αos knockout inhibits the inflammatory response during hepatic I/R injury. **(A)** KEGG pathway enrichment analysis of the major biological pathways. **(B)** Heatmap showing expression of inflammatory genes involved in HIRI. **(C)** TNF-α, IL-1β, IL-6 and MIP-2 levels after liver I/R were measured by ELISA. **(D)** Relative mRNA expression of Tnf-α, Il-1β, Il-6 and Mip-2 after liver I/R was examined by qRT-PCR (n = 5). **(E)** Representative immunofluorescence images of the Ly6G after I/R injury and the quantification of Ly6G-positive cell ratio. The scale bar represents 25 μm. **(F)** Western blot analysis of p-IκBα, IκBα, p-p65, and p65 and the relative band density. n.s. P > 0.05, *P < 0.05, **P < 0.01, ***P < 0.001.

The sterile inflammatory response plays a pivotal role in I/R injury, and the release of cytokines and chemokines is sustained throughout the entire pathophysiological processes of hepatic I/R. Therefore, we performed RNA-seq with I/R challenged liver samples of WT and *Hnf4αos*-KO mice to detect whether *Hnf4αos* can affect liver damage by modulating the inflammatory response. The Kyoto Encyclopedia of Genes and Genomes (KEGG) analysis demonstrated significantly enriched signaling pathways of inflammatory response, in particularly the NF-κB pathway (Fig. 3A). Moreover, heatmap of leading-edge enriched pathways showed that *Hnf4αos* ablation mainly affected the expression of NF-κB signaling related molecules (Fig. 3B). The ELISA and qRT-PCR analysis suggested sham procedure did not induce basal inflammation changes in mice (Fig. 3C and D). *Hnf4αos*-KO mice exhibited less inflammatory cytokine/chemokine (TNF-α, IL-1β, IL-6, and MIP-2) release than WT mice in the I/R model (Fig. 3C and D). In accordance with the data obtained in vivo, the medium collected from the primary *Hnf4αos*-KO hepatocyte culture contained lower levels of cytokines/chemokines (Supplementary Fig. S3A). Tissue MPO activity, an indicator of neutrophil infiltration, was dramatically increased following I/R insult in WT mice. In contrast, *Hnf4αos*-KO mice exhibited less neutrophil accumulation (Supplementary Fig. S3B). Moreover, tissue section immunofluorescence analysis demonstrated fewer Ly6G positive (a neutrophil biomarker) cells when comparing *Hnf4αos*-KO versus WT-I/R mice (Fig. 3E). Gene set enrichment analysis (GSEA) also indicated that *Hnf4αos* could significantly activate the NF-κB signaling pathway (Supplementary Fig. S3C). Subsequently, we found that *Hnf4αos*-KO inhibited the translocation of NF-κB from cytoplasm to nuclear during HIRI (Supplementary Fig. S3D). Further results showed that NF-κB pathway during the I/R process was obviously reversed in the *Hnf4αos*-KO as shown by Western blotting (Fig. 3F). As indicated above, we obtained nearly identical results in *Hnf4α-as1* knockdown and overexpression human L02 hepatocytes (Supplementary Figs. S3E and F).

### 3.4 *Hnf4αos* depletion alleviates apoptosis in hepatic I/R injury

**Fig. 4:**
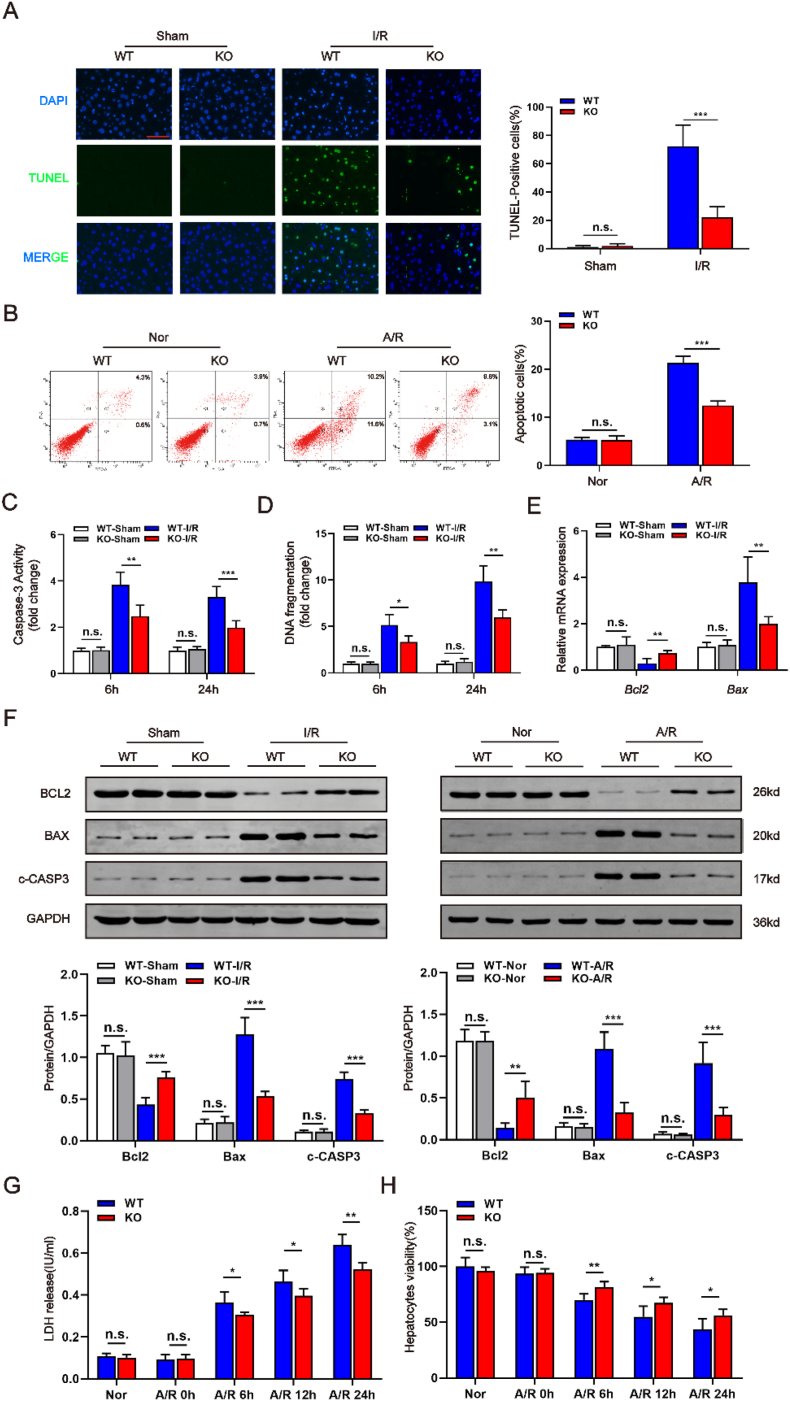
*Hnf4αos* depletion protects hepatocytes from hepatic I/R injury in vivo and in vitro. **(A)** Representative images of liver sections stained by TUNEL and the quantification of the TUNEL-positive cell ratio. The scale bar represents 25 μm. **(B)** Cell apoptosis determined by flow cytometry and the quantification of the apoptotic cells. **(C**–**D)** Caspase-3 activity and DNA fragmentation in mouse liver extracts were determined by ELISA. **(E)** Relative mRNA expression of Bcl2 and Bax. **(F)** Western blot analysis of BCL2, BAX, c-CASP and relative band density. **(G)** LDH release from hepatocytes was measured after A/R treatment. **(H)** Cell viability was determined at different timepoints after A/R treatment by CCK-8 assay. n.s. P > 0.05, *P < 0.05, **P < 0.01, ***P < 0.001.

An excessive inflammatory response inevitably causes cell death, which is accompanied by varying degrees of liver damage [20]. Therefore, we further examined the effects of *Hnf4αos* on cell apoptosis. As expected, the I/R model showed a significant elevation in apoptosis, and we found fewer TUNEL-positive cells in liver tissues from *Hnf4αos*-KO mice than in liver tissues from *Hnf4αos*-WT mice (Fig. 4A). Flow cytometry assay showed that *Hnf4αos* depletion reduced the apoptotic levels of hepatocytes subjected to A/R operation compared to *Hnf4αos*-WT group (Fig. 4B). The results of the caspase-3 activity assay and DNA fragmentation ELISA also suggested dramatic decrease in apoptotic levels with *Hnf4αos* depletion (Fig. 4C and D). As shown by qRT-PCR and Western blot, I/R-induced cell death was markedly blunted in the livers of *Hnf4αos* deficient mice, as evidenced by the expression of apoptotic markers (BCL-2, Bax and cleaved caspase-3) (Fig. 4E and F). Moreover, less LDH was released from *Hnf4αos* deficient hepatocyte cultures than from control hepatocytes (Fig. 4G). The CCK-8 assay results in Fig. 4H showed that *Hnf4αos*-deficiency enhanced cell viability and promoted cell proliferation in *Hnf4αos*-KO mice, compared to control mice. In line with our observations in primary mouse hepatocytes, *Hnf4α-as1*-knockdown in human L02 hepatocytes also alleviated cell apoptosis and *Hnf4α-as1*-overexpression had the opposite effects (Supplementary Fig. S4A).

### 3.5 PGC1α mediates *Hnf4αos* function in hepatic I/R injury

**Fig. 5:**
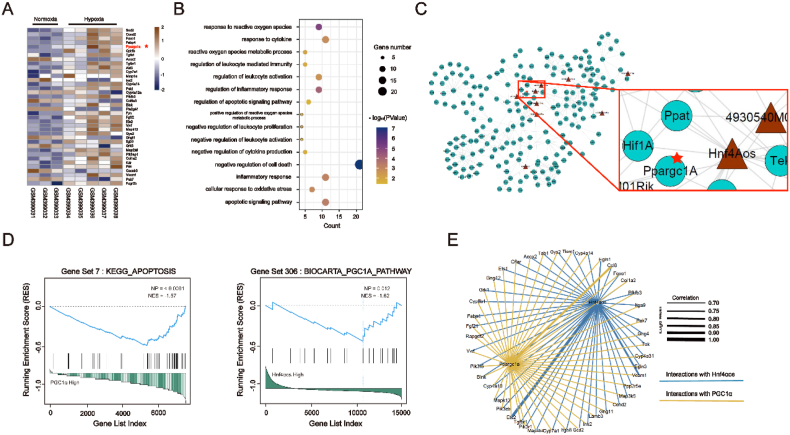
PGC1α is the potential target of *Hnf4αos*. **(A)** Heatmaps generated using the RNA expression of members detected by the DEG (3 mice in the nornoxia group and 5 mice in hypoxia group). **(B)** Gene Ontology (GO) analysis for DEGs that correlated with cell death, oxidative stress and inflammatory response. **(C)** lncRNA-mRNA interaction module of the network indicates that PGC1α and *Hnf4αos* were potentially correlated. Blue nodes represent mRNAs, red nodes represent lncRNAs, and lines indicate interactions. **(D)** GSEA of apoptosis gene signatures in PGC1α enrichment groups and coexpressed genes of PGC1α and *Hnf4αos* function determined by GSEA respectively. **(E)** Gene regulated genes by PGC1α and *Hnf4αos* related to the cell death, oxidative stress and inflammatory response pathways. (For interpretation of the references to color in this figure legend, the reader is referred to the Web version of this article.)

**Fig. 6:**
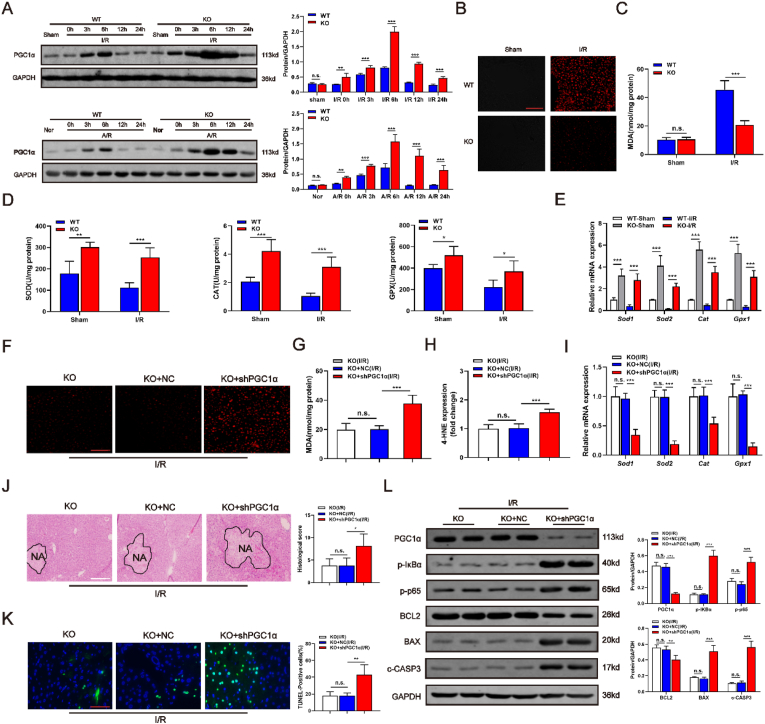
PGC1α mediates *Hnf4αos* function in hepatic I/R injury. **(A)** Western blot analysis of PGC1α in *Hnf4αos*-KO mice and *Hnf4αos*-KO hepatocytes after I/R and A/R treatment and relative band density. **(B)** Representative images of DHE-stained liver cryosections from *Hnf4αos*-KO mice after I/R injury. The scale bar represents 50 μm **(C)** The MDA content after liver I/R injury. **(D)** The activities of SOD, CAT and GPX in the *Hnf4αos*-KO mice after I/R injury. **(E)** The relative expression levels of *Sod1*, *Sod2*, *Cat* and *Gpx1* mRNA. **(F)** Representative images of DHE-stained liver cryosections from *Hnf4αos*-KO mice subjected to Ad-shPGC1α after I/R injury. The scale bar represents 50 μm **(G**–**H)** MDA and 4-HNE contents from *Hnf4αos*-KO mice subjected to Ad-shPGC1α after liver I/R injury. **(I)** The relative expression levels of *Sod1*, *Sod2*, *Cat* and *Gpx1* mRNA from *Hnf4αos*-KO mice subjected to Ad-shPGC1α. **(J)** Representative images of H&E-stained liver sections. The scale bar represents 200 μm. **(K)** Representative images of liver sections stained by TUNEL. The scale bar represents 25 μm. **(L)** Western blot analysis of PGC1α, NF-κB and apoptosis related genes and relative band density. n.s. P > 0.05, *P < 0.05, **P < 0.01, ***P < 0.001.

Based on the GEO data-set (GSE15891), we found the differentially expressed genes (DEGs) (Fig. 5A) are closely related to the regulation of cell death, oxidative and anti-inflammatory response according to the Gene Ontology (GO) analysis (Fig. 5B). Moreover, we established a module by bioinformatic methods to evaluate the potential correlation between the DEGs and differential expressed lncRNAs. The lncRNA-mRNA interaction network (Fig. 5C) surprisingly revealed a close correlation between *Hnf4αos* and PGC1α. We previously reported that PGC1α protected the liver from I/R injury by attenuating hepatocyte death, reducing cytokine/chemokine release and alleviating oxidative stress [13]. GSEA also demonstrated that most genes affected by PGC1α overexpression were involved in the KEGG apoptosis pathway. More importantly, a dramatically negative correlation was found between *Hnf4αos* and PGC1α pathway related molecules (Fig. 5D). Specifically, in Fig. 5E, the module enriched in multiple cell death, oxidative stress and inflammatory pathways also showed a high degree of correlation with *Hnf4αos* and PGC1α expression. Thus, we confirmed an obviously negative association between *Hnf4αos* and PGC1α by Western blot (Fig. 6A). Our previous study found that PGC1α can protect the liver against I/R insult by accelerating the clearance of ROS. Therefore, we hypothesized that *Hnf4αos*-KO ameliorates liver damage in the I/R process by scavenging accumulated ROS. Subsequently, we detected ROS levels by dihydroethidium staining (DHE) and DHE staining showed that in the livers of *Hnf4αos*-KO mice, intracellular concentrations of ROS were markedly decreased compared with those in control mice subjected to I/R operation (Fig. 6B). As indicators of oxidative stress damage, MDA and 4-HNE contents were tested in I/R-treated liver tissues. In line with the results of DHE staining, *Hnf4αos* knockout abrogated the I/R-induced increase in MDA/4-HNE contents and resulted in lower MDA/4-HNE contents (Fig. 6C; Supplementary Fig. S5). Next, we speculated whether the activities of ROS scavenging enzymes were increased, which were induced by *Hnf4αos* knockout-mediated PGC1α upregulation. The hepatic activities of ROS scavenging enzymes (SOD, CAT and GPX) were increased in the KO groups compared with the WT mice following the I/R operation (Fig. 6D). In line with the activities of antioxidative enzymes, the mRNA levels of *Sod1*, *Sod2*, *Cat* and *Gpx1* were dramatically decreased after mice were subjected to the I/R procedure. However, *Hnf4αos*-KO enhanced the expression of those enzymes in the I/R model compared to that in WT mice (Fig. 6E). We then constructed an shPGC1α adenovirus and transferred PGC1α-deficient vectors into *Hnf4αos*-KO mice and primary hepatocytes (Supplementary Figs. S6A and B). Reversibility experiments ensured that PGC1α knockdown abrogated the reduced oxidative stress damage induced by *Hnf4αos*-KO and that *Hnf4αos*-KO-mediated protection against hepatic I/R injury was also reversed by PGC1α deficiency (Fig. 6F–L; Supplementary Figs. S6C–E).

### 3.6 *Hnf4αos* promotes the stability of *Hnf4α* mRNA

**Fig. 7:**
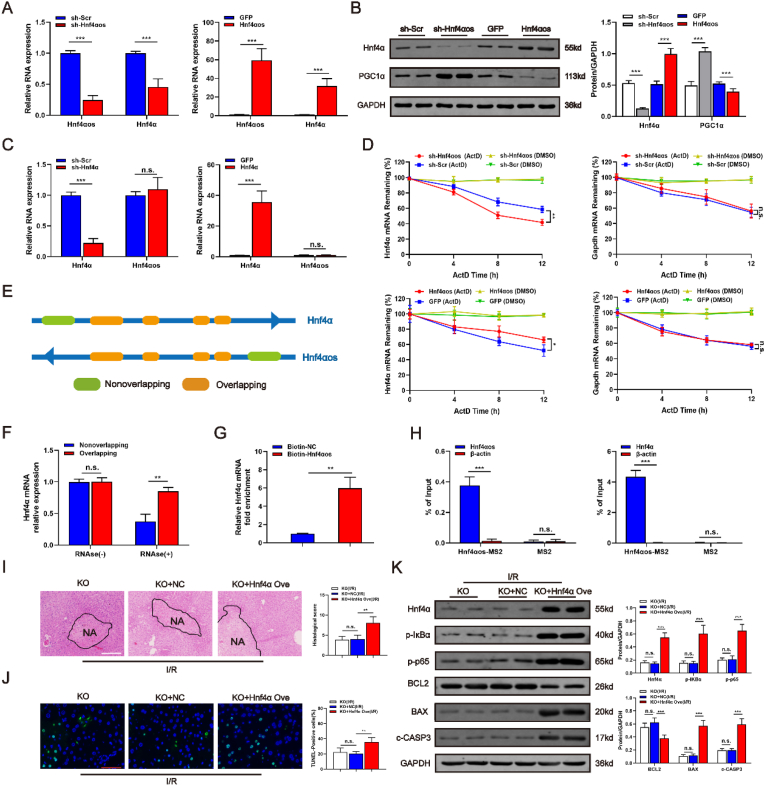
*Hnf4αos* promotes the stability of *Hnf4α* mRNA. **(A)** The relative expression levels of Hnf4α mRNA in Ad-*Hnf4αos* and Ad-sh*Hnf4αos* cells. **(B)** Western blot analysis of Hnf4α in Ad-*Hnf4αos* and Ad-sh*Hnf4αos* cells and relative band density. **(C)** The relative expression levels of *Hnf4αos* RNA in Ad-Hnf4α and Ad-shHnf4α cells. **(D)** After treatment with ActD (5 g/ml), the stability of *Hnf4α* and *Gapdh* mRNA in the cells transfected with Ad-*Hnf4αos*, Ad-sh*Hnf4αos* and the respective control vectors was determined by qRT-PCR at different timepoints. **(E)** Schematic representation of the *Hnf4αos*/*Hnf4α* locus. **(F)***Hnf4α* mRNA levels measured by qRT-PCR followed by ribonuclease protection assay. **(G)** The interaction between *Hnf4α* and biotin-*Hnf4αos* was detected by a biotin RNA pulldown assay followed by qRT-PCR. **(H)** The interaction between *Hnf4α* and *Hnf4αos* was detected by TRAP assay. **(I)** Representative images of H&E-stained liver sections from *Hnf4αos*-KO mice subjected to Ad-Hnf4α after liver I/R injury. The scale bar represents 200 μm. **(J)** Representative images of liver sections stained by TUNEL. The scale bar represents 25 μm. **(K)** Western blot analysis of Hnf4α, NF-κB and apoptosis related genes and relative band density. n.s. P > 0.05, *P < 0.05, **P < 0.01, ***P < 0.001.

To determine how *Hnf4αos* manipulates hepatocyte viability by regulating PGC1α, we further conducted an in-depth study of the structural features of *Hnf4αos*. *Hnf4αos* is a natural antisense transcript (NAT) of *Hnf4α* known for its transcriptional regulation of several hepatic genes. As reported previously, antisense lncRNAs are used to bind to the respective sense strand mRNA to form a duplex strand, which enhances the stability of the latter mRNA [[21], [22], [23]]. We further explored the mRNA and protein levels of Hnf4α accompanied by *Hnf4αos* alteration. As shown in Fig. 7A and B, downregulated *Hnf4αos* expression significantly decreased the mRNA and protein levels of Hnf4α. Conversely, *Hnf4αos* overexpression enhanced the expression levels of Hnf4α. Then, we constructed Hnf4α overexpression and Hnf4α knockdown adenovirus vectors (Supplementary Figs. S7A–B). However, the variations in Hnf4α expression had no effects on the *Hnf4αos* transcript (Fig. 7C). To determine whether *Hnf4αos* regulated the stability of *Hnf4α* mRNA, we performed an RNA stability assay. *Hnf4αos*-KO and *Hnf4αos-*overexpressing hepatocytes were treated with actinomycin D (ActD) to inhibit mRNA transcription. qRT-PCR analysis showed that *Hnf4αos* downregulation markedly shortened the half-life of *Hnf4α* mRNA and that *Hnf4αos* overexpression elevated the level of *Hnf4α* mRNA (Fig. 7D). These findings indicate that *Hnf4αos* positively regulates *Hnf4α* mRNA expression.

In the case of the *Hnf4αos/Hnf4α* pair, complementarity was noted in both transcripts (Fig. 7E). To determine the existence of a sense-antisense RNA duplex, a ribonuclease protection assay (RPA) was performed and showed that the complementary region was protected from degradation by RNase, indicating an RNA duplex between lncRNA *Hnf4αos* and *Hnf4α* mRNA (Fig. 7F). Furthermore, the biotin-labeled RNA pulldown assay and tagged RNA affinity purification (TRAP) assay revealed a strong interaction between *Hnf4αos* and endogenous *Hnf4α* mRNA (Fig. 7G and H). We noticed that enhanced expression of Hnf4α worsened liver injury (Fig. 7I–K, Supplementary Figs. S7C and E) and activated a sterile inflammatory response (Fig. 7K, Supplementary Fig. S7D), as evidenced by more severe tissue necrosis and cytokine/chemokine release, which could be ameliorated by *Hnf4αos*-KO. Collectively, these data support the conclusion that *Hnf4αos* increased the stability of *Hnf4α* mRNA, which was modulated by the duplex of *Hnf4αos/Hnf4α*.

### 3.7 Hnf4α mediates the suppressive effect of miR-23α on PGC1α

**Fig. 8:**
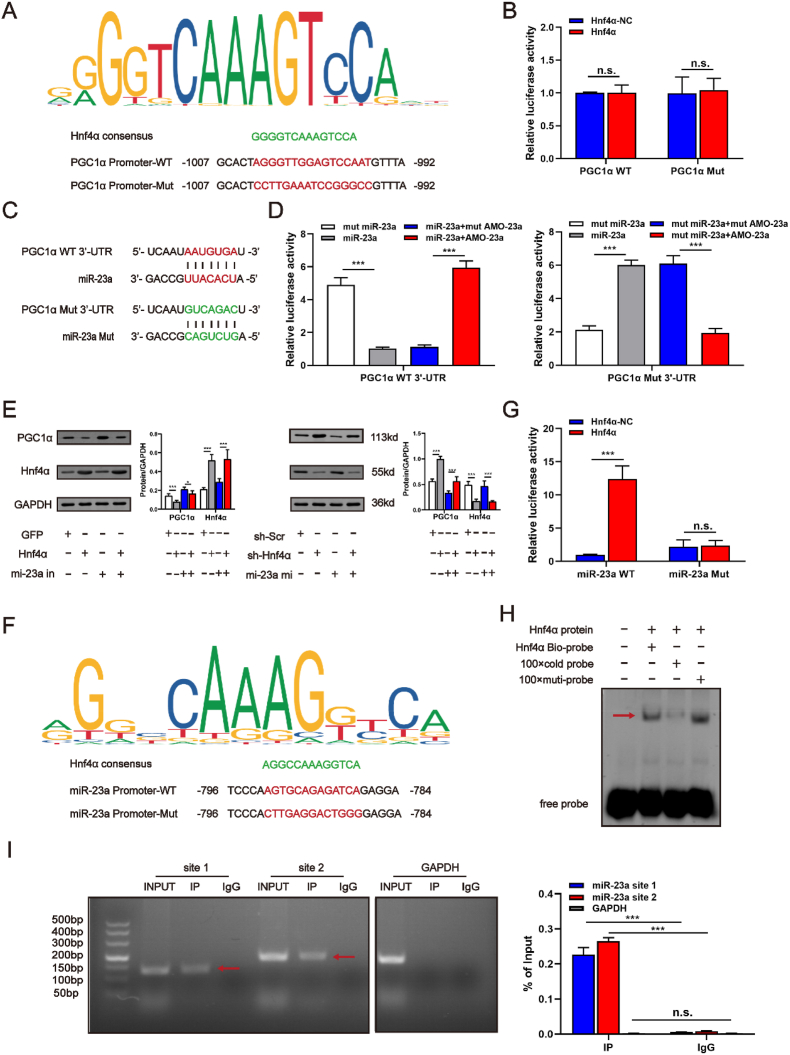
Hnf4α enhances miR-23a transcription by binding to its promoter region. **(A)** Hnf4α consensus and its potential binding sites on the PGC1α promoter. **(B)** The results of the luciferase reporter assay. **(C)** Complementary WT and Mut sequence alignment of miR-23a and PGC1α. **(D)** The results of the luciferase reporter assay. **(E)** Western blot analysis of PGC1α and Hnf4α with the transfection of Ad-Hnf4α, Ad-shHnf4α, miR-23a inhibitor and miR-23a mimics and relative band density. **(F)** Hnf4α consensus and its potential binding sites on the miR-23a promoter. **(G)** The results of the luciferase reporter assay. **(H)** EMSA was performed with nuclear extracts and radiolabeled probes encompassing the candidate Hnf4α-binding sequence on the miR-23a promoter. **(I)** ChIP assay showing the binding of Hnf4α to the miR-23a promoter. n.s. P > 0.05, *P < 0.05, **P < 0.01, ***P < 0.001.

To further confirm the exact mechanism through which *Hnf4αos* regulated PGC1α expression, we speculated that Hnf4α exerted a directive transcriptional inhibitory effect on PGC1α by acting as a transcription factor (TF). In support of our hypothesis, we analyzed the PGC1α promoter sequences using the UCSC, JASPAR, SWISSREGULON and PROMO algorithms and surprisingly found that the promoter region of PGC1α has a candidate binding site for TF-Hnf4α (Fig. 8A). The luciferase reporter assay demonstrated no relationship between Hnf4α and the transcriptional activity of PGC1α (Fig. 8B). Numerous reports have shown that miR-23a is a key regulator of PGC1α expression [[24], [25], [26]], and we found a physical interaction between miR-23a and PGC1α through the miRDB, RNAinter, TargetScan and miRmap databases (Fig. 8C). The luciferase reporter assay confirmed that miR-23a was a negative regulator of PGC1α (Fig. 8D). Then, we performed qRT-PCR to detect the RNA level of miR-23a between *Hnf4αos* and *Hnf4α* (Supplementary Fig. S8). To confirm that miR-23a contributes to the function of PGC1α in hepatic I/R injury, we constructed miR-23a mimics and inhibitors. Western blot analysis showed that miR-23a and Hnf4α deficiency dramatically upregulated the protein levels of PGC1α, conversely, miR-23a/Hnf4α overexpression suppressed PGC1α protein expression (Fig. 8E). Given that the considerable lncRNA *Hnf4αos* enhances the stability of Hnf4α, we speculated whether TF-Hnf4α mediated the transcription of miR-23a and subsequently attenuated the expression of PGC1α. Intriguingly, based on the prediction by the database, we found that Hnf4α binding sites in the promoter of miR-23a and revealed that the transcription of miR-23a was dramatically activated by TF-Hnf4α (Fig. 8F and G). Consistently, nuclear extracts were obtained and used for an electrophoretic mobility shift assay (EMSA), and the results identified marked DNA-protein binding activity in mouse primary hepatocytes (Fig. 8H). Furthermore, chromatin immunoprecipitation (ChIP) assays provided evidence for the direct interaction of Hnf4α with the miR-23a promoter (Fig. 8I). Together, the data above showed that a significant interaction between the promoter region of miR-23a and TF-Hnf4α.

**Fig. 9:**
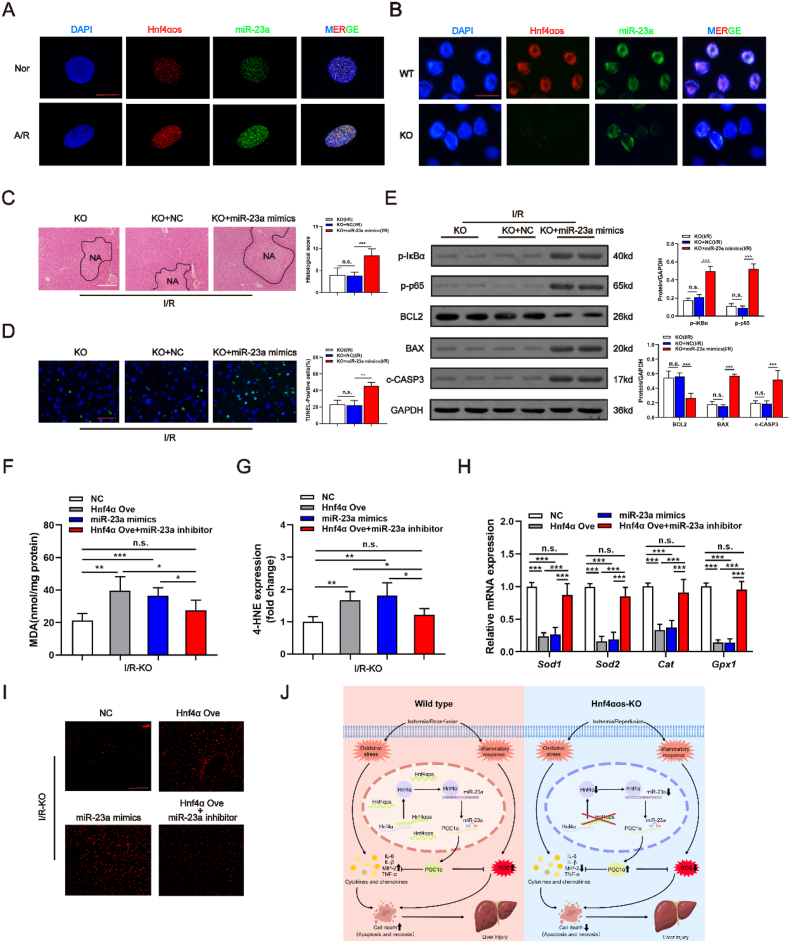
miR-23a exacerbates liver damage and oxidative stress induced I/R injury. **(A)** The cellular expression of *Hnf4αos* and miR-23a was analyzed by dual RNA-FISH after A/R treatment. The scale bar represents 10 μm. **(B)** The cellular expression of *Hnf4αos* and miR-23a was analyzed by Dual RNA-FISH in *Hnf4αos*-KO hepatocytes. The scale bar was 20 μm. **(C)** Representative images of H&E-stained liver sections from *Hnf4αos*-KO mice subjected to miR-23a mimics after liver I/R injury and the quantification of histological score. The scale bar represents 200 μm. **(D)** Representative images of liver sections stained by TUNEL after I/R injury and the quantification of the TUNEL-positive cell ratio. The scale bar was 25 μm. **(E)** Western blot analysis of NF-κB and apoptosis related genes and relative band density. **(F–I)** miR-23a inhibitor reverses the levels of oxidative stress induced by Hnf4α overexpression. **(J)** Mechanism involved in protective effects of *Hnf4αos*-KO after liver subjected to I/R insult. n.s. P > 0.05, *P < 0.05, **P < 0.01, ***P < 0.001.

As shown by confocal microscopy examination, both *Hnf4αos* and miR-23a levels were increased in A/R-treated cells compared with normoxic cells by dual-RNA FISH detection (Fig. 9A). Moreover, the primary hepatocytes subjected to *Hnf4αos*-KO exhibited almost no red/green fluorescence signals, while the fluorescence signals of WT-cells were much stronger (Fig. 9B). Further experiments validated that miR-23a overexpression attenuated the protective effects of *Hnf4αos*-KO on liver I/R injury (Fig. 9C–E and Supplementary Figs. S9A–C). Importantly, both Hnf4α and miR-23a also abrogated the antioxidative effects induced by *Hnf4αos*-KO, and miR-23a knockdown suppressed the oxidative activation of Hnf4α overexpression as demonstrated by Fig. 9F–I. Finally, miR-23a deficiency ameliorated liver damage and the inflammatory response induced by Hnf4α overexpression (Supplementary Figs. S9D–G). These data suggest that Hnf4α mediates the suppressive effect of miR-23α on PGC1α.

## 4 Discussion

HIRI is the most important effector in liver surgery, particularly in liver transplantation. In the present study, we found a differentially expressed lncRNA – *Hnf4αos* during HIRI progression in both human and mouse models. Knocking out *Hnf4αos* in hepatocytes significantly suppressed the oxidative stress – induced hepatic injury and inhibited the inflammatory response during HIRI both in vitro and in vivo. Using the integrated approaches of bioinformatic analysis, we identify the potential interaction of *Hnf4αos* and PGC1α, and *Hnf4αos* facilitated the RNA decay of PGC1α by ceRNA function. Thus, *Hnf4αos* could be a promising therapeutic target of HIRI.

Oxidative stress – induced liver injury plays dominant roles during HIRI progression. The production of ROS caused by the oxidative stress response triggers peroxidation reactions, which activate the apoptotic pathway and decrease hepatocyte viability in hepatic I/R injury [27,28]. Therefore, regulation of ROS metabolism is expected to have the potential to effectively protect the liver against I/R injury. In the current study, we found that the reduced *Hnf4αos* level exhibited a significant antioxidative effects by regulating the balance of ROS scavenging and accumulation systems. Considering that PGC1α is key mediator of ROS metabolism [13,18], we speculated that *Hnf4αos* regulated the oxidative stress in HIRI by targeting PGC1α. To verify our hypothesis, *Hnf4αos*-KO mice were generated. *Hnf4αos* deficiency in vivo and in vitro reduces the degree of hepatic I/R and improves hepatic function in mice by PGC1α elevation-mediated ROS scavenging compared to WT conditions.

Functionally, lncRNAs can bind not only to proteins but also to DNA and RNA, rendering lncRNAs a crucial factor in protein-nucleic acid/nucleic acid-nucleic acid networks. Several studies, including ours, have provided strong evidence that NATs regulate the expression of their sense protein-coding mRNAs [21,22]. Here, our findings demonstrated that *Hnf4αos* and *Hnf4α* formed an RNA – RNA duplex and further promoted *Hnf4α* mRNA stability, which consequently enhanced the protein level of Hnf4α as shown by Western blot.

Hnf4α generally functions as a transcription factor in the liver and has been reported to play prominent roles in cell proliferation, cell differentiation, lipid metabolism and gluconeogenesis [[29], [30], [31], [32]]. Several studies have revealed that Hnf4α is a key regulator in inhibiting hepatocyte proliferation. Walesky et al. found that hepatocyte-specific depletion of Hnf4α induced increased levels of cell proliferation. Further microarray analysis demonstrated that a significant number of genes known to be promitogenic were upregulated by Hnf4α-deficiency [33]. It has been reported that Hnf4α promoted the transcriptional activity of ASK1, which is a typical proapoptotic mediator in MAPK pathway [34] and Mai et al. confirmed the antiapoptotic potential of Hnf4α-deficiency in endometriosis [35]. Furthermore, a study related to viral hepatitis suggested that knocking down Hnf4α markedly inhibited HBV RNA transcripts and respective DNA replication intermediates, which played a key role in delaying the progression of HBV-induced hepatitis [36]. Although Hnf4α exerts antineoplastic activity in HCC, Hnf4α was reported to act as an oncogene in gastrointestinal adenocarcinomas and pancreatic cancer [37,38], indicating multiple roles of Hnf4α. In our study, we found that Hnf4α served as a TF binding site in the promoter of miR-23a and subsequently further activated its transcription, showing antiproliferative and proapoptotic effects in HIRI accompanied by *Hnf4αos* depletion.

Although our clinical data showed a downward trend of *Hnf4α-as1* in patients who underwent partial liver resections, *Hnf4α* and miR-23a were also downregulated simultaneously during I/R insult, which may be due to the species differences between humans and mice [39] (Supplementary Fig. S10). More importantly, *Hnf4α-as1* deficiency also exerted hepatoprotective effects on the process of HIRI, and *Hnf4α-as1* overexpression had the opposite effects. Consequently, from this perspective, clinical therapeutic strategies targeting *Hnf4α-as1* can be reasonably established.

## 5 Conclusions

In conclusion, our findings demonstrate a strategy to manipulate PGC1α activity by *Hnf4αos*. Specifically, *Hnf4αos*-mediated stabilization of *Hnf4α* mRNA reverses the protective effect of PGC1α by upregulating miR-23a expression, leading to a reduction in the scavenging levels of ROS and exacerbation of hepatic I/R injury. Thus, targeting *Hnf4α-as1* may provide potential clinical benefits for liver I/R injury.

## Declaration of competing interest

No potential conflicts of interest were disclosed.

## Funding

This work was jointly supported by grants from the Outstanding Youth Training Fund from Academician Yu Weihan of 10.13039/100010722Harbin Medical University (2014), Harbin Medical University Postgraduate Innovation and Practical Research Project (YJSCX2020-28HYD), Scientific Foundation of the First Affiliated Hospital of 10.13039/100010722Harbin Medical University (2019L01, HYD2020JQ0007, HYD2020JQ0011), Heilongjiang Postdoctoral Foundation (LBH-Z11066, LBH-Z12201 and LBH-Q17097), 10.13039/501100002858China Postdoctoral Science Foundation (2012M510990, 2012M520769 and 2013T60387), 10.13039/501100005046Natural Science Foundation of Heilongjiang Province of China (LC2018037) and the 10.13039/100014717National Natural Scientific Foundation of China (81100305, 81470876 and 81270527).

## Data Availability

Data will be made available on request.

## References

[1] Sun P., Lu Y.X., Cheng D., Zhang K., Zheng J., Liu Y. (2018). Monocyte chemoattractant protein‐induced protein 1 targets hypoxia‐inducible factor 1α to protect against hepatic ischemia/reperfusion injury. Hepatology.

[2] Bamboat Z.M., Balachandran V.P., Ocuin L.M., Obaid H., Plitas G., DeMatteo R.P. (2010). Toll‐like receptor 9 inhibition confers protection from liver ischemia–reperfusion injury. Hepatology.

[3] Wang Y., Hylemon P.B., Zhou H. (2021). Long non‐coding RNA H19: a key player in liver diseases. Hepatology.

[4] Kung J.T., Colognori D., Lee J.T. (2013). Long noncoding RNAs: past, present, and future. Genetics.

[5] Li D., Liu X., Zhou J., Hu J., Zhang D., Liu J. (2017). Long noncoding RNA HULC modulates the phosphorylation of YB‐1 through serving as a scaffold of extracellular signal–regulated kinase and YB‐1 to enhance hepatocarcinogenesis. Hepatology.

[6] Yang J.J., Yang Y., Zhang C., Li J., Yang Y. (2020). Epigenetic silencing of LncRNA ANRIL enhances liver fibrosis and HSC activation through activating AMPK pathway. J. Cell Mol. Med..

[7] Huang F., Liu H., Lei Z., Li Z., Zhang T., Yang M. (2020). Long noncoding RNA CCAT1 inhibits miR‐613 to promote nonalcoholic fatty liver disease via increasing LXRα transcription. J. Cell. Physiol..

[8] Kawai J., Shinagawa A., Shibata K., Yoshino M., Itoh M., Ishii Y. (2001). Functional annotation of a full-length mouse cDNA collection. Nature.

[9] Katayama S., Tomaru Y., Kasukawa T., Waki K., Nakanishi M., Nakamura M. (2005). Antisense transcription in the mammalian transcriptome. Science.

[10] Mootha V.K., Lindgren C.M., Eriksson K.-F., Subramanian A., Sihag S., Lehar J. (2003). PGC-1α-responsive genes involved in oxidative phosphorylation are coordinately downregulated in human diabetes. Nat. Genet..

[11] Lin J., Handschin C., Spiegelman B.M. (2005). Metabolic control through the PGC-1 family of transcription coactivators. Cell Metabol..

[12] Wang C., Dong L., Li X., Li Y., Zhang B., Wu H. (2021). The PGC1α/NRF1-MPC1 axis suppresses tumor progression and enhances the sensitivity to sorafenib/doxorubicin treatment in hepatocellular carcinoma. Free Radic. Biol. Med..

[13] Wang C., Li Z., Zhao B., Wu Y., Fu Y., Kang K. (2021). PGC-1α protects against hepatic ischemia reperfusion injury by activating PPARα and PPARγ and regulating ROS production. Oxid. Med. Cell. Longev..

[14] Wang X., Mao W., Fang C., Tian S., Zhu X., Yang L. (2018). Dusp14 protects against hepatic ischaemia–reperfusion injury via Tak1 suppression. J. Hepatol..

[15] Wang D., Ma Y., Li Z., Kang K., Sun X., Pan S. (2012). The role of AKT1 and autophagy in the protective effect of hydrogen sulphide against hepatic ischemia/reperfusion injury in mice. Autophagy.

[16] Yan Z.Z., Huang Y.P., Wang X., Wang H.P., Ren F., Tian R.F. (2019).

[17] Pan S., Liu L., Pan H., Ma Y., Wang D., Kang K. (2013).

[18] Li D., Wang C., Ma P., Yu Q., Gu M., Dong L. (2018).

[19] Faghihi M.A., Modarresi F., Khalil A.M., Wood D.E., Sahagan B.G., Morgan T.E. (2008).

[20] Bhogal R.H., Sutaria R., Afford S.C. (2011). Hepatic liver ischemia/reperfusion injury: processes in inflammatory networks—a review. Liver Transplant..

[21] Faghihi M.A., Modarresi F., Khalil A.M., Wood D.E., Sahagan B.G., Morgan T.E. (2008). Expression of a noncoding RNA is elevated in Alzheimer's disease and drives rapid feed-forward regulation of β-secretase. Nat. Med..

[22] Jadaliha M., Gholamalamdari O., Tang W., Zhang Y., Petracovici A., Hao Q. (2018). A natural antisense lncRNA controls breast cancer progression by promoting tumor suppressor gene mRNA stability. PLoS Genet..

[23] Li B., Hu Y., Li X., Jin G., Chen X., Chen G. (2018). Sirt1 antisense long noncoding RNA promotes cardiomyocyte proliferation by enhancing the stability of Sirt1. J. Am. Heart Assoc..

[24] Sun L.-Y., Wang N., Ban T., Sun Y.-H., Han Y., Sun L.-L. (2014). MicroRNA-23a mediates mitochondrial compromise in estrogen deficiency-induced concentric remodeling via targeting PGC-1α. J. Mol. Cell. Cardiol..

[25] Wang C., Li Q., Wang W., Guo L., Guo C., Sun Y. (2015). GLP-1 contributes to increases in PGC-1α expression by downregulating miR-23a to reduce apoptosis. Biochem. Biophys. Res. Commun..

[26] Du J., Hang P., Pan Y., Feng B., Zheng Y., Chen T. (2019). Inhibition of miR-23a attenuates doxorubicin-induced mitochondria-dependent cardiomyocyte apoptosis by targeting the PGC-1α/Drp1 pathway. Toxicol. Appl. Pharmacol..

[27] Abu‐Amara M., Yang S.Y., Tapuria N., Fuller B., Davidson B., Seifalian A. (2010). Liver ischemia/reperfusion injury: processes in inflammatory networks—a review. Liver Transplant..

[28] Zwacka R.M., Zhou W., Zhang Y., Darby C.J., Dudus L., Halldorson J. (1998). Redox gene therapy for ischemia/reperfusion injury of the liver reduces AP1 and NF-κB activation. Nat. Med..

[29] Chen L., Vasoya R.P., Toke N.H., Parthasarathy A., Luo S., Chiles E. (2020). HNF4 regulates fatty acid oxidation and is required for renewal of intestinal stem cells in mice. Gastroenterology.

[30] Thakur A., Wong J.C., Wang E.Y., Lotto J., Kim D., Cheng J.C. (2019). Hepatocyte nuclear factor 4‐alpha is essential for the active epigenetic state at enhancers in mouse liver. Hepatology.

[31] Wu H., Reizel T., Wang Y.J., Lapiro J.L., Kren B.T., Schug J. (2020). A negative reciprocal regulatory axis between cyclin D1 and HNF4α modulates cell cycle progression and metabolism in the liver. Proc. Natl. Acad. Sci. USA.

[32] Walesky C., Apte UJGe (2015).

[33] Walesky C., Gunewardena S., Terwilliger E.F., Edwards G., Borude P., Apte UJAJoP -G. (2013).

[34] Jiang C.-F., Wen L.-Z., Yin C., Xu W.-P., Shi B., Zhang X. (2016).

[35] Mai H., Liao Y., Luo S., Wei K., Yang F., Shi HJJoc (2021).

[36] He F., Chen E.Q., Liu L., Zhou T.Y., Liu C., Cheng X. (2012).

[37] Pan J., Silva T.C., Gull N., Yang Q., Plummer J.T., Chen S. (2020).

[38] Sun Q., Xu W., Ji S., Qin Y., Liu W., Hu Q. (2019).

[39] Ruan X., Li P., Chen Y., Shi Y., Pirooznia M., Seifuddin F. (2020).

