# Supplementary material for: LncRNA Hnf4αos exacerbates liver ischemia/reperfusion injury in mice via Hnf4αos/Hnf4α duplex-mediated PGC1α suppression
Source: Redox Biol. 2022 Oct 6;57:102498. doi: 10.1016/j.redox.2022.102498 (PMC9576992; doi:10.1016/j.redox.2022.102498)
Supplement: Multimedia component 1 [file mmc1.docx]

**Supplementary Materials and Methods.**

**SiRNA and plasmids transfection**

For transient transfection, L02 cells (1×10^5^ cells) were seeded in 24 well plates, and 50mM siRNA (RiboBio, Guangzhou, China) and 2 μg plasmids (GeneChem, Shanghai, China) were transfected into cells with Lipofectamine 2000 (Invitrogen, Carlsbad, CA).

**Quantitative real-time PCR and Western blot**

Total RNA was extracted from cells and tissues using an AxyPrep Multisource Total RNA Miniprep Kit (Axygen Scientific, Inc., USA) according to the manufacturer’s instructions and reverse transcribed into cDNA using a qPCR RT Kit (TOYOBO, Shanghai, China) after RNA quantification. Real-time PCR was performed using THUNDERBIRD SYBR qPCR Mix (TOYOBO, Shanghai, China) on an ABIPRISM 7500HT instrument (Applied Biosystems). The expression levels of the target mRNA/microRNA were normalized to the glyceraldehyde-3-phosphate dehydrogenase (GAPDH) and U6 expression level and was determined according to the 2-ΔΔCt method. The primer sequences used in the study were listed in Supplementary Table S4. To examine protein levels, whole-cell lysates were extracted with RIPA extraction reagent (Beyotime, Beijing, China). A 50 μg protein sample was fractionated on a 10% SDS-polyacrylamide gel and transferred to a PVDF membrane (Merck Millipore Ltd., Germany). The membrane was blocked with 5% skim-milk in PBST for 1h at room temperature and then incubated with the primary antibodies and horseradish peroxidase-conjugated suitable secondary antibodies. Finally, the membrane was incubated with IRDye 800CW secondary antibodies (LI-COR, USA) (1:10,000) at room temperature for 1 hour and the Odyssey® Imaging System (LI-COR, USA) was used to visualize and analyse the proteins. Primary antibodies against the following target proteins were used: PGC1α (merk) (1:2,000)( ST1202)， p-IκBα (Cell Signaling Technology)(1:1,000)(2859), IκBα (Cell Signaling Technology)(1:1,000)(4814)，p-P65 (Cell Signaling Technology)(1:1,000)(3033)，P65 (Cell Signaling Technology)(1:1,000)(6956)，Bax (Cell Signaling Technology)(1:1,000)(2772), Bcl-2 (Cell Signaling Technology)(1:1,000)(3498), c-CASP3 (Cell Signaling Technology)(1:500)(9661), Hnf4α (Santa Cruz Biotechnology)(1:2,000)(sc-374229), GAPDH (Kangcheng)(1:8,000)(KC-5G4).

**Adenovirus and oligonucleotides transfection**

Adenovirus vectors for *Hnf4αos* and Hnf4α overexpression (Ad-*Hnf4αos*; Ad-Hnf4α) and downregulation (Ad-sh*Hnf4αos*; Ad-shHnf4α) were constructed and obtained from GeneChem Corporation (Shanghai, China). The corresponding empty vectors (Ad-GFP, and Ad-shScramble) were used as negative controls (GeneChem, Shanghai, China). The adenoviral vector for the expression of PGC1α (Ad-PGC-1α) or GFP alone (Ad-GFP) was a kind gift from Daniel P. Kelly (Washington University School of Medicine, St. Louis, MO). Oligonucleotides for mimics, inhibitors and respective control were purchased from RiboBio (Guangzhou, China). Primary hepatocytes were transfected with Ad-*Hnf4αos*/Ad-GFP (MOI=80), Ad-sh*Hnf4αos*/Ad-shScramble (MOI=80), Ad-Hnf4α/Ad-GFP (MOI=80), Ad-shHnf4α/Ad-shScramble (MOI=80), Ad-PGC1α/Ad-GFP (MOI=80), Ad-shPGC1α/Ad-shScramble (MOI=80) and mimics (60 nmol/L), inhibitor (100 nmol/L) and their corresponding controls (100 nmol/L) using Lipofectamine2000 (Invitrogen, Carlsbad, CA). Information on all the target sequences was provided in Supplementary Table S5.

**Liver damage assessment**

The levels of alanine aminotransferase (ALT) and aspartate aminotransferase (AST) in sera were measured using an autobiochemical analyzer (Toshiba, TBA-20FR). The extent of necrosis in the ischemic lobes was assessed using hematoxylin and eosin (H&E) staining. The results of the assessment were expressed as the sum of the individual score grades, including 0 (no findings), 1 (mild), 2 (moderate), to 3 (severe) for each of the following six parameters: cytoplasmic color fading, vacuolization, nuclear condensation, nuclear fragmentation, nuclear fading, and erythrocyte stasis.

**Terminal dUTP nick-end labeling (TUNEL) assay**

We performed a TUNEL assay (Roche, Shanghai, China) according to the manufacturer’s instructions to detect apoptosis levels in vivo and quantified TUNEL-positive cells in ten randomly selected × 800 high-power fields under microscopy.

**Malondialdehyde (MDA) determination**

The levels of MDA in liver tissues were determined by the thiobarbituric acid method using an assay kit from Nanjing Jiancheng Bioengineering Institute (Nanjing, China) and the liver homogenate protein was measured by the CBB method. The MDA content was expressed as nmol per mg protein and calculated as follows:

MDA concentration (nmol/mg prot) = [(measuring tube OD−control tube OD)/(standard tube OD−blank tube OD)]×standard substance concentration÷protein concentration

**Hepatic 4-hydroxynonenal (4-HNE) content**

Lipid peroxides are unstable indicators of oxidative stress in cells that decompose to form more complex and reactive compounds such as 4-HNE, which has been shown to be capable of binding to proteins and forming stable HNE adducts. The hepatic HNE content was determined using a kit (Nanjing, China). In brief, BSA or hepatic tissue extracts (10 μg/mL) were adsorbed onto a 96-well plate for 12 h at 4 °C. HNE adducts present in the sample or standard were probed with an anti-HNE antibody, followed by an HRP-conjugated secondary antibody. The HNE-protein adduct content in an unknown sample was determined by comparison with a standard curve.

**Isolation of nuclear and cytoplasmic RNA**

Nuclear and cytoplasmic RNA fractions from mouse primary hepatocytes were isolated and purified with the PARIS Kit Protein & RNA Isolation System (Invitrogen, Lithuania). RNA levels were detected by quantitative real-time PCR. 18s (a specific cytoplasmic RNA marker) and U6 (a nuclear RNA marker) were examined in the two fractions as controls.

**Enzyme-linked immunosorbent assay (ELISA)**

The serum and cell culture medium levels of TNF-α, IL-6, IL-1β and MIP-2 were measured with enzyme-linked immunosorbent assay (ELISA) kits (R&D Systems, Minneapolis, MN) according to the manufacturer’s instructions.

**Myeloperoxidase (MPO) activity assay**

MPO activity was detected with a Myeloperoxidase assay kit (Nanjing Jiancheng, China) according to the manufacturer’s instructions.

**Cell viability and apoptosis assay**

Cell viability was assessed by the Cell Counting Kit-8 (CCK-8, Dojindo Molecular Technologies, CK04–13) according to the instructions of the manufacturer. Cell cytotoxicity was assessed by measuring lactate dehydrogenase (LDH) release into the medium by necrotic cells using an LDH Cytotoxicity Assay Kit (Promega, Madison, WI, USA).

**Caspase-3 activity assay**

Caspase-3 activity was performed using Caspase-3 Cellular Activity Assay Kit (Beyotime, China). Liver tissue sample and cell lysis were used according to the manufacturer’s instruction.

**DNA fragmentation ELISA**

The quantitative determinations of cytoplasmic histone-associated-DNA-fragmentation (mono and oligonucleosomes) due to cell death were measured using the Cell Death Detection ELISA kit (Roche Diagnostics GmbH, Indianapolis, IN), according to the manufacturer’s protocol.

**Measurement of the activities of SOD, CAT and GPX.**

The activities of SOD, CAT and GPX were determined with commercially available assay kits from Nanjing Jiancheng Bioengineering Institute (Nanjing, China). Briefly, homogenate protein concentration in the liver was measured by the CBB method. The T-SOD activity was measured as that which will inhibit the rate of oxidation of hydroxylamine by 50% in a coupled system, using xanthine and xanthine oxidase at 37 °C in 1.0 mg/mL protein concentration of liver tissue homogenate. The CAT activity was measured by the rate of disappearance of H_2_O_2_ at 240 nm and expressed as micromoles of H_2_O_2_ decomposed per minute per gram of liver tissue. The GPX activity is the amount of enzyme that will oxidize 1 μmol/L GSH in the reaction system at 37 °C per minute in 1 mg of liver tissue. The activities of these parameters were expressed as units of nitrite per mg protein. All samples were measured in triplicate.

**RNA stability assay**

Primary hepatocytes were treated with Actinomycin D (ActD) (MedChemExpress) (5 μg/ml) and collected at different time points after adenovirus transfection. Total RNA extracts were obtained for further qRT-PCR.

**Biotin RNA pulldown assays**

Biotinylated *Hnf4αos* and a negative control (Gapdh) were transfected into AML12 cells. An AML12 whole cell extract was obtained followed by streptavidin-mediated RNA pull down. Finally, we isolated total RNA from suspended beads for further qRT-PCR to analyze potential RNA interactions.

**Tagged RNA affinity purification (TRAP)**

According to the manufacturer’s instructions (BersinBio, Guangzhou, China), Hnf4α sequences were connected with plasmids and further transfected into AML12 cells. Then, the cells were collected and lysed. Hnf4α mRNA was captured by magnetic beads coated with probes and lncRNAs bound to Hnf4α were precipitated. The lncRNAs were collected for further quantification PCR.

**Chromatin immunoprecipitation assay (ChIP)**

We performed chromatin immunoprecipitation (ChIP) assays using chromatin immunoprecipitation kits (Millipore, Billerica, MA, USA) according to the manufacturer’s instructions. The abundance of Hnf4α at the miR-23a promoter was measured by real-time PCR. The primers are listed in Supplementary Table S6.

**RNA-FISH assay**

Dual RNA-FISH assays were performed with BersinBio (Guangzhou，China) as described. Cells were washed in PBS and fixed in 4% paraformaldehyde for 10 min at room temperature. Then the cells were permeabilized in PBS containing 0.5% Triton X-100 at 4 ℃ for 5 min, washed with PBS three times for 5 min, and prehybridized at 37 ℃ for 30 min before hybridization. Then, anti-*Hnf4αos* and anti-*miR-23a* oligodeoxynucleotide probes were used in the hybridization solution at 37 ℃ overnight in the incubator. The next day, the cells were counterstained with DAPI and imaged using a confocal laser-scanning microscope (Carl Zeiss, Germany).

**Animal study**

Ad-*Hnf4αos*, Ad-sh*Hnf4αos*, Ad-Hnf4α, Ad-shHnf4α, Ad-PGC1α, Ad-shPGC1α and respectively negative control adenovirus vectors were delivered to mice via mouse tail vein injection with 1×10^9^ TCID50/mouse for 72 h before the mouse subjected to liver I/R operation as previously described. For rescue experiments, adenovirus vectors of PGC1α knockdown (1×10^9^ TCID50/mouse) and Hnf4α overexpression (1×10^9^ TCID50/mouse), oligonucleotides (10 mg/kg) for miRNA mimics and inhibitors were given a tail-vein injection.

**Bioinformatic datasets**

To characterize differentially expressed genes in the liver, the expression profile was downloaded from the Gene Expression Omnibus (GEO) database (GSE15891). For the expression profile (20103700), the probe sets were reannotated using the Affymetrix Mouse Genome 430 2.0 Array platform by referring to Li et al. 's steps (32164040). Log2 transformation was performed for raw gene expression values. Then, fold changes (FCs) and p values were computed for each gene. The statistical significance of differential expression was computed for gene expression profiles in datasets by the R/Bioconductor software package (17988400). The genes were designated differentially expressed genes (DEGs), and the cutoff criterion was a p value < 0.05.

**Analysis of functions and co-expression network of DEGs**

To understand the functions of DEGs, the co-expression network was constructed based on expression profiles by calculating the Pearson correlation coefficient (PCC>0.8). The R package ‘SubpathwayMiner’ was also employed to reconstruct the co-expression network, resulting in 1043 DEGs and 34 lncRNAs (19706733). Co-expression modules were detected using Cytoscape v3.6.1 software with function ‘jActiveModules’. Additionally, all DEGs in co-expression network were used to analyze Kyoto Encyclopedia of Genes and Genes (KEGG) pathway enrichment (22543366). The oxidative stress or inflammatory response pathways (p-value < 0.05) were considered as significantly enriched and retained for following downstream analyses.

**Gene set enrichment analysis (GSEA).**

To further investigate the biological pathways involved in liver injury pathogenesis, we performed a gene set enrichment analysis (GSEA). Firstly, the co-expression genes of PGC1α and *Hnf4αos* were downloaded from Genefriends database (https://genefriends.org/) (25361971), *Hnf4αos*’s correlation obtained by ensemble transcript id (ENSMUST00000141329) in database. Secondly, an overlap gene list of the DEGs and co-expression genes was constructed as Gene sets for PGC1α and Hnf4aos respectively. The phenotype labeled with GSEA that Pearson correlation coefficient was per gene for ranking. Finally, C2 gene sets in MSigDB (molecular signature database) v7.0 were used for GSEA.

**Bioinformatic analysis**

The process of bioinformatic analysis is illustrated in Fig.1B: we selected the top 10 differentially expressed lncRNAs and the 124 mRNAs associated oxidative stress/inflammatory response/apoptosis. The pairs of |pcc| ＞ 0.9 were extracted from lncRNAs-mRNAs networks. Finally, the 82 pairs associated oxidative stress/inflammatory response/apoptosis were retained for further research.

The process of bioinformatic analysis is illustrated in Fig. 5: (i) construction of the lncRNAs-mRNAs network; (ii) the mRNAs of the biological process (BP) annotations; (iii) identification of the module of network and (iv) inference of the PGC1α and Hnf4αos determined by GSEA respectively. The method is described in detail as follows:

(i) Construction of the lncRNAs-mRNAs network

For gene expression profile, Genes were performed Log2 transformation with raw expression values and fold change (FC) were computed for each gene. The process of lncRNAs referenced by the Zhao et. al[1]. Statistical significance for the differentially expressed genes (DEGs) was computed by R/Bioconductor software package (p<0.05). For the DEGs, Pearson correlation coefficients were calculated in the data sets (Pcc>0.8 or Pcc<-0.8). A global lncRNAs-mRNAs network was finally constructed by the SubpathwayMiner software package and optimized by random walk algorithm.

(ii) The mRNAs of the biological process (BP) annotations

We conducted the ontology (GO) biological process (BP) of DAVID. The BP function annotations were only considered the oxidative stress, the anti-inflammatory response and apoptosis of pathways. Meanwhile, the intersections were obtained between the genes of annotations and the DEGs.

(iii) Identification of the module of lncRNAs-mRNAs network

The jActiveModules plugin of Cytoscape was used to identify the modules from lncRNAs-mRNAs network[2]. Subsequently, the modules were retained for containing the relationships between PGC1α and Hnf4αos.

(iv)Inference of PGC1α and Hnf4αos determined by GSEA respectively.

Target genes of PGC1α and Hnf4αos were downloaded from GeneFriends and intersected with all genes in the expression profile respectively[3]. They were ordered in a ranked list based on Pcc values. Moreover, to inference the enrichment functions of PGC1α and Hnf4αos, GSEA was used to determine if target genes were statistically significant to related to the oxidative stress, the anti-inflammatory response and apoptosis pathways[4]. Additionally, co-expression genes of PGC1α and Hnf4αos were extracted from lncRNAs-mRNAs network separately base on the oxidative stress, the anti-inflammatory response and apoptosis pathways. Finally, the set of PGC1α and Hnf4αos performed the intersection operation.

**Reference**:

1. Liao Q, Liu C, Yuan X, Kang S, Miao R, Xiao H, et al: Large-scale prediction of long non-coding RNA functions in a coding–non-coding gene co-expression network. 2011; 39:3864-3878.

2. Ideker T, Ozier O, Schwikowski B, Siegel AFJB: Discovering regulatory and signalling circuits in molecular interaction networks. 2002; 18:S233-S240.

3. van Dam S, Cordeiro R, Craig T, van Dam J, Wood SH, de Magalhães JPJBg: GeneFriends: an online co-expression analysis tool to identify novel gene targets for aging and complex diseases. 2012; 13:1-14.

4. Subramanian A, Tamayo P, Mootha VK, Mukherjee S, Ebert BL, Gillette MA, et al: Gene set enrichment analysis: a knowledge-based approach for interpreting genome-wide expression profiles. 2005; 102:15545-15550.

**Table S1.**

The result of risk lncRNAs

| **Entrezgene ID** | **LncRNA Symbol** | **Score** | **Rank** |
| --- | --- | --- | --- |
| 72818 | 2810471M01Rik | 0.003280456 | 5 |
| 73874 | 4930419G24Rik | 0.002820827 | 8 |
| 319894 | E330017L17Rik | 0.002144114 | 19 |
| 80515 | Rnf227 | 0.002128913 | 20 |
| 70523 | 5730420D15Rik | 0.00206254 | 22 |
| 67194 | 2700038G22Rik | 0.00203209 | 25 |
| 68314 | Hnf4aos | 0.001906443 | 32 |
| 100504714 | Gm16793 | 0.001786102 | 39 |
| 112414 | 4930540M05Rik | 0.001701639 | 44 |
| 76311 | 1110019D14Rik | 0.001622571 | 57 |
| 67303 | 3110045C21Rik | 0.001568567 | 63 |
| 73183 | 5430402O13Rik | 0.001525873 | 71 |
| 100036520 | Gata5os | 0.001482903 | 80 |
| 111975 | Igf2os | 0.001426396 | 88 |
| 320977 | A330023F24Rik | 0.001335613 | 110 |
| 100503019 | Gm16551 | 0.001303037 | 122 |
| 100503518 | Gm11827 | 0.001246889 | 139 |
| 654820 | G530011O06Rik | 0.001155235 | 181 |
| 19296 | Pvt1 | 0.001098394 | 209 |
| 414095 | B130034C11Rik | 0.001087086 | 212 |
| 320163 | 4930525G20Rik | 0.001038078 | 239 |
| 102633312 | 1700001J04Rik | 0.001034886 | 242 |
| 100504121 | 4930525G20Rik | 0.001022166 | 252 |
| 636791 | Gm9866 | 0.000985679 | 274 |
| 638247 | 9530082P21Rik | 0.00098401 | 275 |
| 67524 | 1700095A21Rik | 0.000983654 | 276 |
| 75115 | 4930509E16Rik | 0.00087852 | 332 |
| 74937 | 4930481B07Rik | 0.000853311 | 352 |
| 100042464 | 2610203C20Rik | 0.000842949 | 359 |
| 70487 | 5730403I07Rik | 0.000840605 | 360 |
| 100043489 | 1300002E11Rik | 0.000822499 | 370 |
| 100502835 | LOC100502835 | 0.000754813 | 420 |
| 666737 | 4632427E13Rik | 0.000677828 | 493 |
| 77127 | A930001A20Rik | 0.000652473 | 526 |

**Table S2.**

Expression profile of lncRNAs in NONCODE

| **No.** | **LncRNAs** | **Heart** | **Hippocampus** | **Liver** | **Lung** | **Spleen** | **Thymus** |
| --- | --- | --- | --- | --- | --- | --- | --- |
| **1** | **2810471M01Rik** | **√** | **×** | **×** | **√** | **√** | **×** |
| **2** | **4930419G24Rik** | **×** | **×** | **×** | **×** | **×** | **×** |
| **3** | **E330017L17Rik** | **×** | **×** | **×** | **√** | **√** | **√** |
| **4** | **Rnf227** | **--** | **--** | **--** | **--** | **--** | **--** |
| **5** | **5730420D15Rik** | **√** | **√** | **×** | **√** | **√** | **×** |
| **6** | **2700038G22Rik** | **√** | **×** | **×** | **×** | **×** | **×** |
| **7** | **Hnf4αos** | **×** | **×** | **√** | **×** | **√** | **×** |
| **8** | **Gm16793** | **×** | **×** | **×** | **×** | **√** | **√** |
| **9** | **4930540M05Rik** | **--** | **--** | **--** | **--** | **--** | **--** |
| **10** | **1110019D14Rik** | **√** | **√** | **×** | **√** | **√** | **√** |
| **11** | **3110045C21Rik** | **×** | **×** | **×** | **×** | **√** | **×** |
| **12** | **5430402O13Rik** | **√** | **×** | **×** | **×** | **√** | **×** |
| **13** | **Gata5os** | **×** | **×** | **×** | **×** | **√** | **√** |
| **14** | **Igf2os** | **√** | **×** | **×** | **√** | **√** | **√** |

**√**  **Expression of lncRNA in mouse tissues × No expression of lncRNA in mouse tissues -- No item of lncRNA**

**Table S3.**

Detailed clinical information of the hemangioma patients.

| **Case No.** | **Sex** | **Age (y)** | **BMI**  **(kgs/m^2^)** | **Diagnosis** | **Ischemia**  **time (min)** | **Reperfusion**  **time (min)** |
| --- | --- | --- | --- | --- | --- | --- |
| **1** | F | 38 | 25.8 | Hemangioma | 15 | 120 |
| **2** | F | 51 | 25.4 | Hemangioma | 22 | 90 |
| **3** | F | 32 | 25.2 | Hemangioma | 15 | 80 |
| **4** | F | 52 | 20.3 | Hemangioma | 16 | 76 |
| **5** | F | 66 | 25.7 | Hemangioma | 15 | 140 |
| **6** | F | 33 | 21.4 | Hemangioma | 17 | 97 |
| **7** | F | 53 | 19.7 | Hemangioma | 15 | 156 |
| **8** | F | 56 | 23.0 | Hemangioma | 16 | 124 |
| **9** | F | 45 | 22.0 | Hemangioma | 15 | 157 |
| **10** | F | 57 | 22.9 | Hemangioma | 15 | 61 |
| **11** | F | 49 | 24.2 | Hemangioma | 15 | 60 |
| **12** | F | 51 | 24.2 | Hemangioma | 15 | 120 |

**Table S4.**

List of primers of qRT-PCR used in this study.

| Gene | Forward Primer | Reverse Primer |
| --- | --- | --- |
| *Hnf4αos* | TCCGGATATGGCAAACTGAT | AGCTTCCTTCAGTGCCTTCACTT |
| *Hnf4α-as1* | TCCTTTAGCGGCTTCCAAGG | GCCTGTCTTCAAGTCACCGA |
| PGC1α | CTTGGTCAAAAGCGCACTAAATC | AAAATCAGGTGTACGTTTCTGGT |
| Hnf4α(Nonoverlap) | AACACGATGCCCTCTCACCTCAGCAA | CTTGACGATGGTGGTGATGGCTCCTG |
| Hnf4α(Overlap) | ACAGTTTGAAAGAGAGGAAGGCAGAG | CCCTACCCACGCCTTAGCCCCCTGTC |
| Tnf-α | CCCTCACACTCAGATCATCTTCT | GCTACGACGTGGGCTACAG |
| IL-1β | GCAACTGTTCCTGAACTCAACT | ATCTTTTGGGGTCCGTCAACT |
| IL-6 | TAGTCCTTCCTACCCCAATTTCC | TTGGTCCTTAGCCACTCCTTC |
| Mip-2 | CCAACCACCAGGCTACAGG | GCGTCACACTCAAGCTCTG |
| Bax | TGAGCGAGTGTCTCCGGCGAAT | GCACTTTAGTGCACAGGGCCTTG |
| Bcl2 | TGGTGGACAACATCGCCCTGTG | GGTCGCATGCTGGGGCCATATA |
| Sod1 | AACCAGTTGTGTTGTCAGGAC | CCACCATGTTTCTTAGAGTGAGG |
| Sod2 | CCAAAGGAGAGTTGCTGGAG | GAACCTTGGACTCCCACAGA |
| Catalase | AGCGACCAGATGAAGCAGTG | TCCGCTCTCTGTCAAAGTGTG |
| Gpx1 | AGTCCACCGTGTATGCCTTCT | GAGACGCGACATTCTCAATGA |
| Gapdh | AGTGGCAAAGTGGAGATT | GTGGAGTCATACTGGAACA |
| miR-23a | GGATCACATTGCCAGGGAT | CAGTGCGTGTCGTGGAGT |
| U6 | CTCGCTTCGGCAGCACA | AACGCTTCACGAATTTGCGT |
| 18S | TGGTGGAGCGATTTGTCTGGTT | CAATCTCGGGTGGCTGAACG |

**Table S5.**

shRNA target sequence and miRNA mimics and inhibitor sequence.

|  | Target sequence |
| --- | --- |
| sh*Hnf4αos* | CCACCACGCAGACAAGAAATT |
| shPGC1α | GGTGGATTGAAGTGGTGTAGA |
| shHnf4α | CCGGGCACCAATGTCATTGTTGCTACTCGAGTAGCAACAATGACATTGGTGCTTTTT |
| miR-23a mimics | AUCACAUUCCAGGGAUUCC |
| miR-23a inhibitor | GGAAAUCCCUGGCAAUGUGAU |

**Table S6.**

Primers flanking the Hnf4α site on the miR-23a promoter

| Primers flanking the Hnf4α site on the miR-23a promoter: | |
| --- | --- |
| Sense（site 1） | 5′-TGCCTCAGTTTCCTCAATGC-3′ |
| anti-sense | 5′- TGCACCCCAACAATAACACC-3′ |
| Primers flanking the Hnf4α site on the miR-23a promoter: | |
| Sense（site 2） | 5′- TGGTGCATTCGGAAACCTTG-3′ |
| anti-sense | 5′- TGTGACTGGCATCAAATCCC-3′ |
| Primers for GAPDH (negative control): | |
| sense | 5′-CTGCTGAAGTGCTCCCTACC-3′ |
| anti-sense | 5′-CCCTTTTCTGCCTTCCTACC-3′ |

**Supplementary Figures**


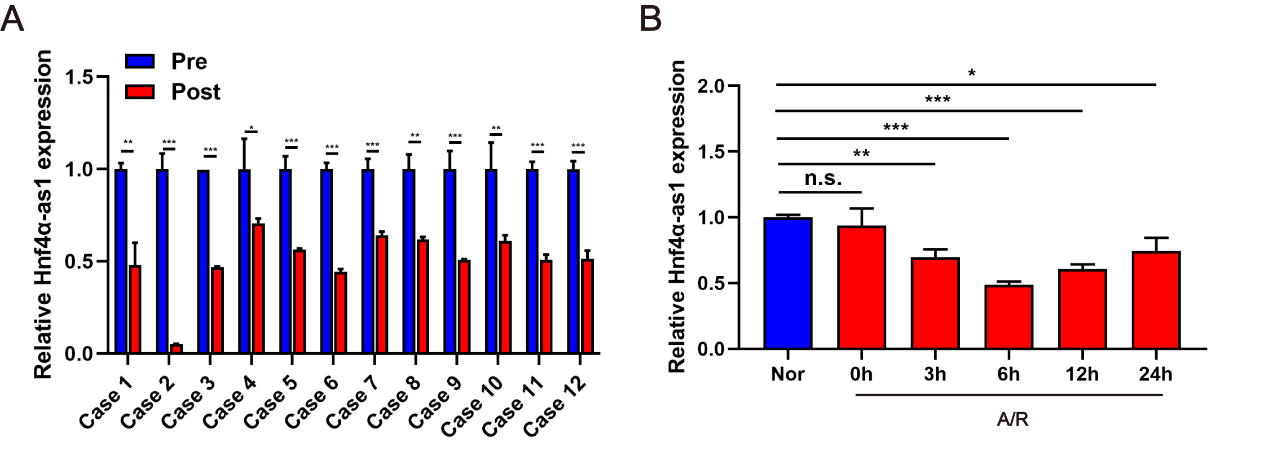


**Fig. S1 *Hnf4α-as1* was evaluated in clinical human I/R samples and human L02 hepatocytes. (A)** *Hnf4α-as1* expression was assessed by qRT-PCR in the livers of individuals underwent I/R surgery. **(B)** *Hnf4α-as1* expression was assessed by qRT-PCR in L02 cell line after A/R treatment. * P < 0.05, ** P < 0.01, *** P < 0.001.


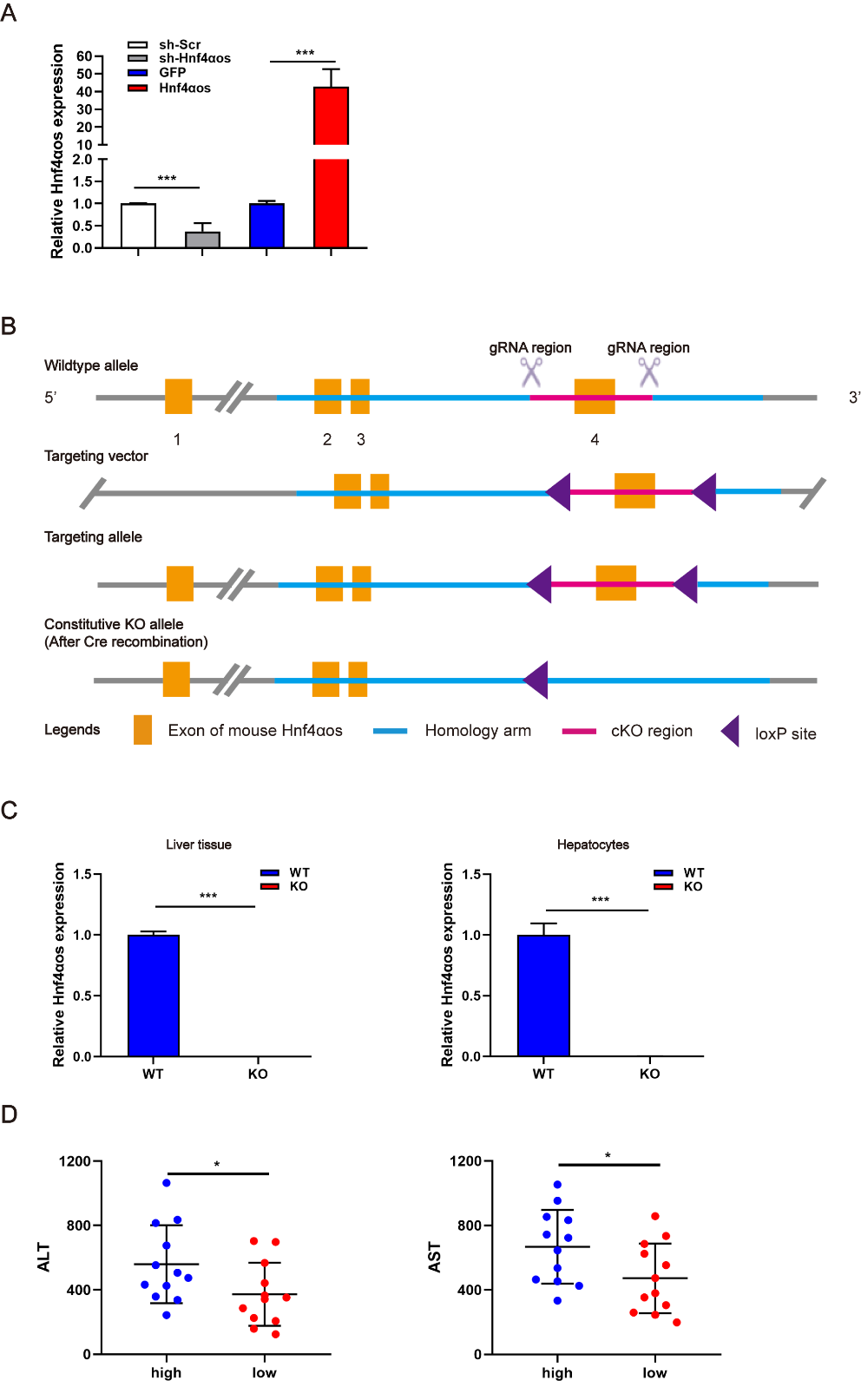


**Fig. S2 Transfection efficiency after adenovirus transfection and establishment of the *Hnf4αos* genic mice. (A)** Transfection efficiency after adenovirus transfection detected by qRT-PCR. **(B)** Schematic workflow of the establishment of hepatocyte-specific *Hnf4αos*-KO mouse strain. **(C)** The RNA expression level of *Hnf4αos* in the indicated liver tissues and hepatocytes. **(D)** Serum levels of aminotransferases (ALT and AST) were detected in patients after partial hepatectomy. * P < 0.05, ** P < 0.01, *** P < 0.001.


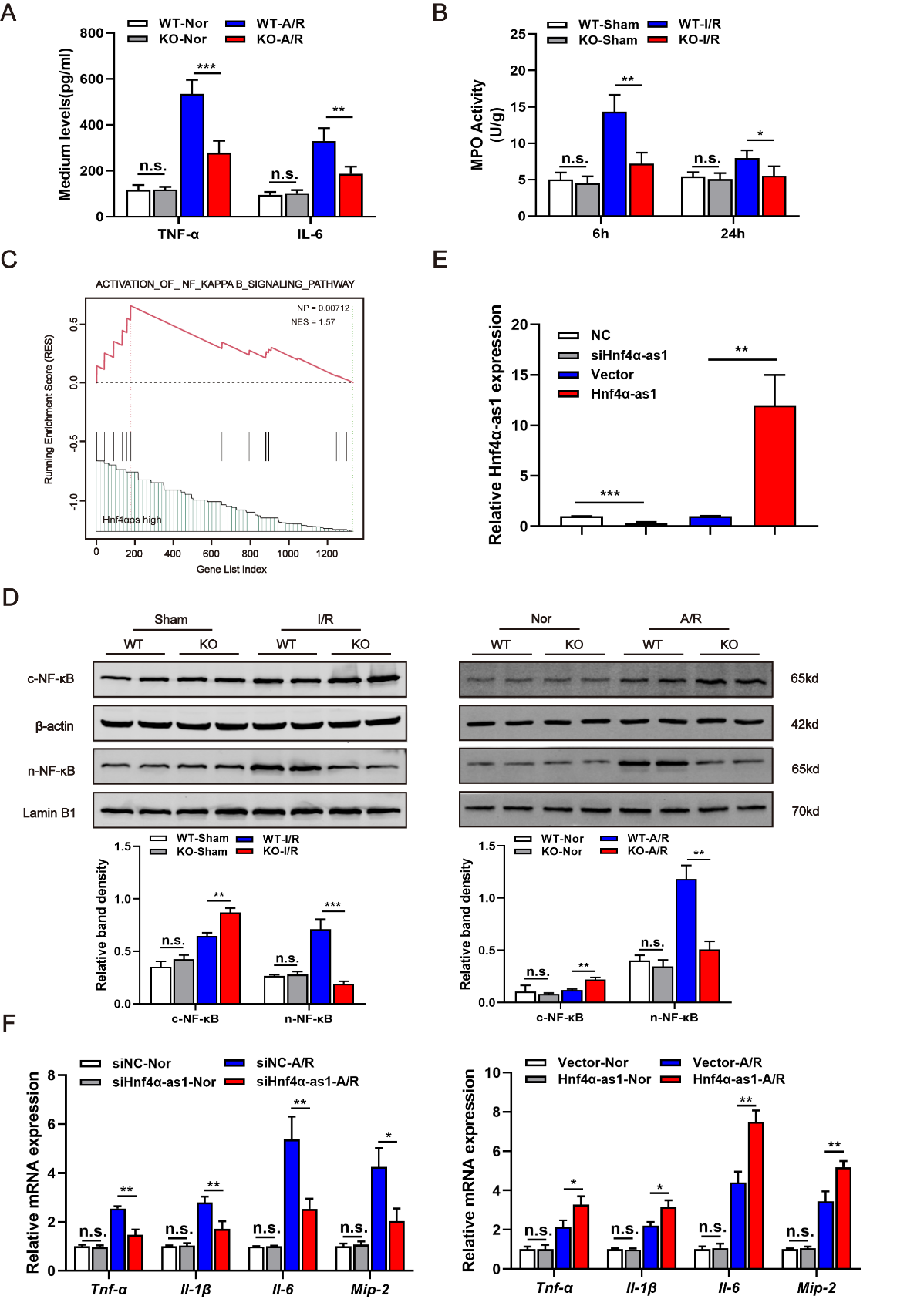


**Fig. S3 *Hnf4α-as1* aggravates inflammatory response in liver I/R injury. (A)** TNF-α and IL-6 levels in the medium of hepatocytes after A/R treatment (n=5). **(B)** MPO activity measured in liver extract after I/R (n=5). **(C)** GSEA analysis indicates a significant change of NF-κB signaling induced by *Hnf4αos*. **(D)** Western blot analysis of cytoplasm NF-κB, nuclear NF-κB and the relative band density. **(E)** The transfection efficiency of si-*Hnf4α-as1* and *Hnf4α-as1* overexpression plamids transfection. **(F)** *Hnf4α-as1* knockdown decreased the release of cytokine/chemokine and *Hnf4α-as1* overexpression increased the release of cytokine/chemokine. * P < 0.05, ** P < 0.01, *** P < 0.001.


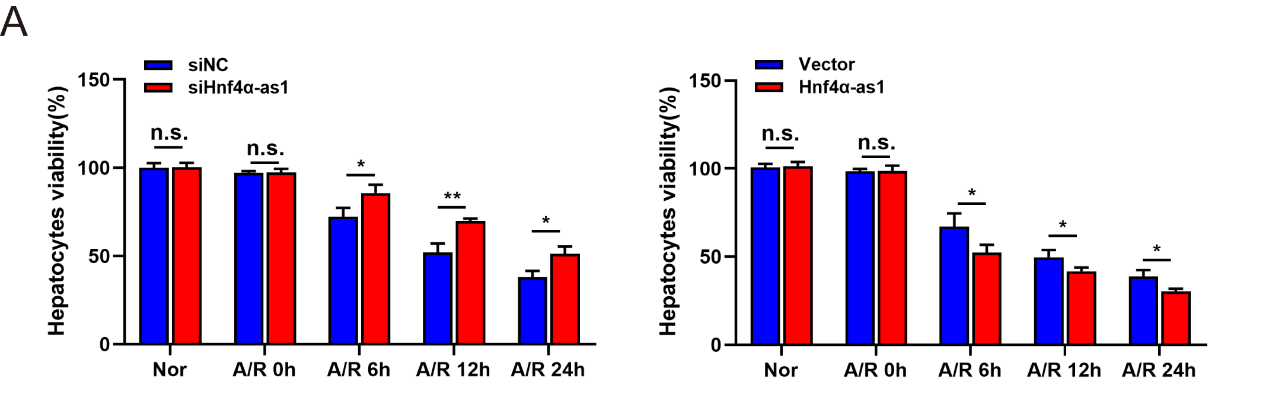


**Fig. S4 *Hnf4α-as1* exacerbates hepatocytes apoptosis in liver I/R injury. (A)** *Hnf4α-as1* knockdown decreased the hepatocytes viability and *Hnf4α-as1* overexpression increased the hepatocytes viability. * P < 0.05, ** P < 0.01, *** P < 0.001.


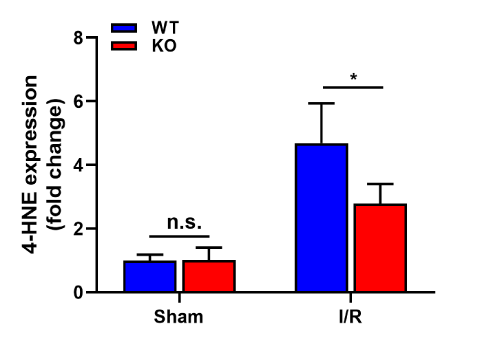


**Fig. S5 The 4-HNE content after liver I/R injury from *Hnf4αos*-KO mice and WT mice.** n.s. P > 0.05, * P < 0.05.


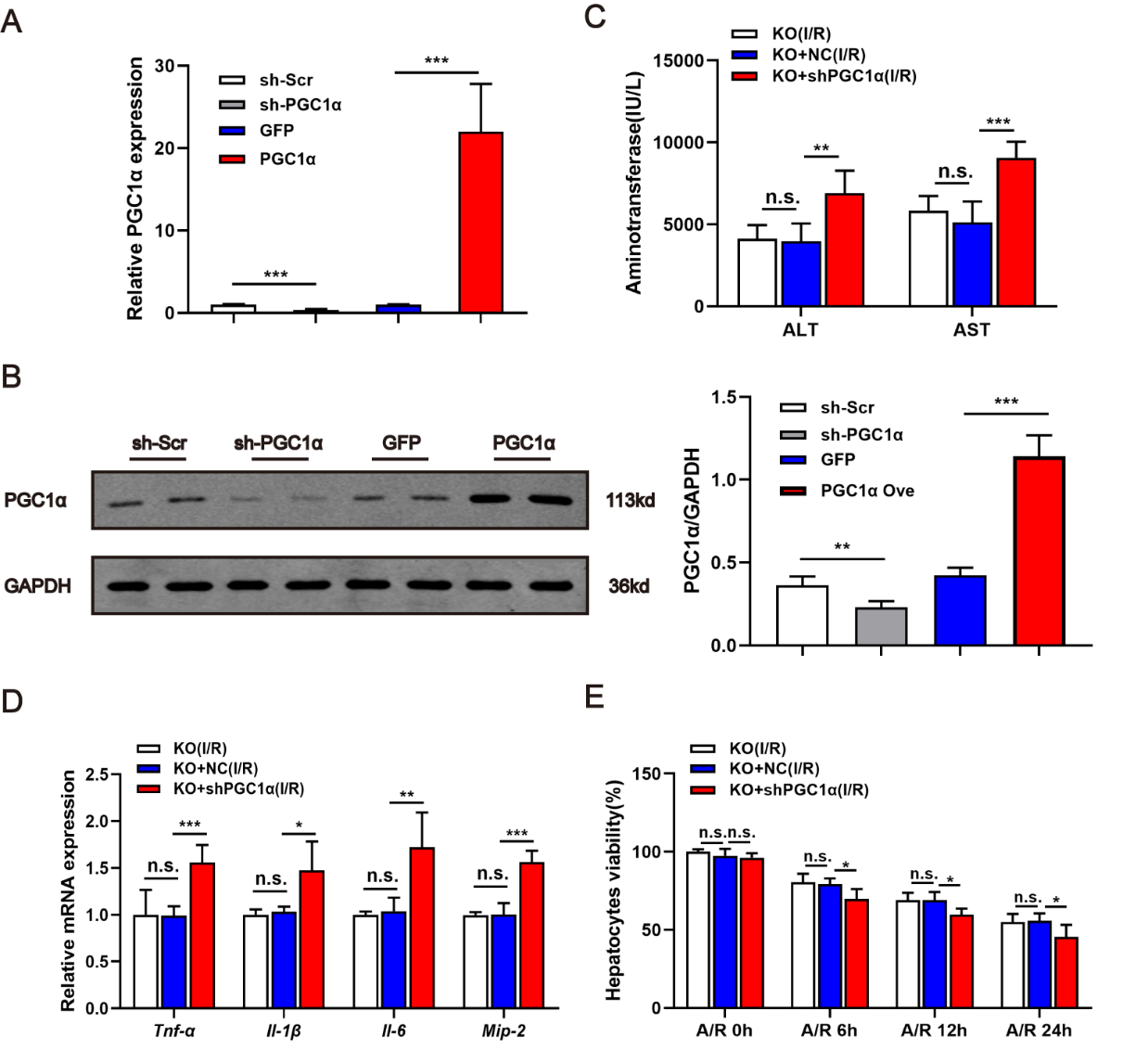


**Fig. S6 Transfection efficiency after adenovirus transfection and PGC1α knockdown deteriorates liver I/R injury. (A-B)** Transfection efficiency after adenovirus transfection detected by qRT-PCR and western blot. **(C)** PGC1α knockdown increases serum ALT and AST contents in *Hnf4αos*-KO mice. **(D)** PGC1α knockdown increases cytokines/chemokines release in *Hnf4αos*-KO mice. **(E)** PGC1α knockdown inhibits cell viability in *Hnf4αos*-KO mice. n.s. P > 0.05, * P < 0.05, ** P < 0.01, *** P < 0.001.


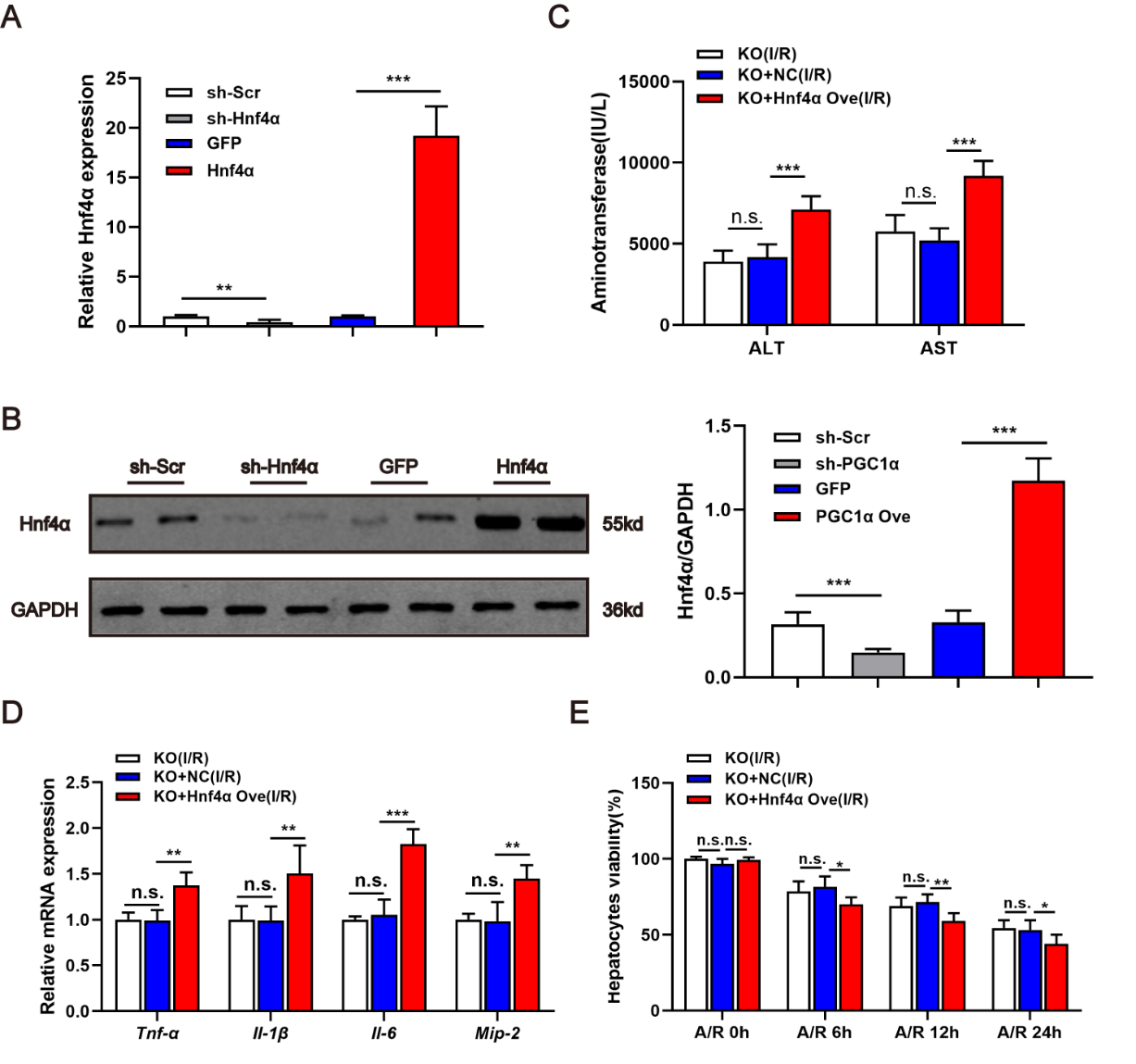


**Fig. S7 Transfection efficiency after adenovirus transfection and Hnf4α overexpression deteriorates liver I/R injury. (A-B)** Transfection efficiency after adenovirus transfection detected by qRT-PCR and western blot. **(C)** Hnf4α overexpression increases serum ALT and AST contents in *Hnf4αos*-KO mice. **(D)** Hnf4α overexpression increases cytokines/chemokines release in *Hnf4αos*-KO mice. **(E)** Hnf4α overexpression inhibits cell viability in *Hnf4αos*-KO mice. n.s. P > 0.05, * P < 0.05, ** P < 0.01, *** P < 0.001.


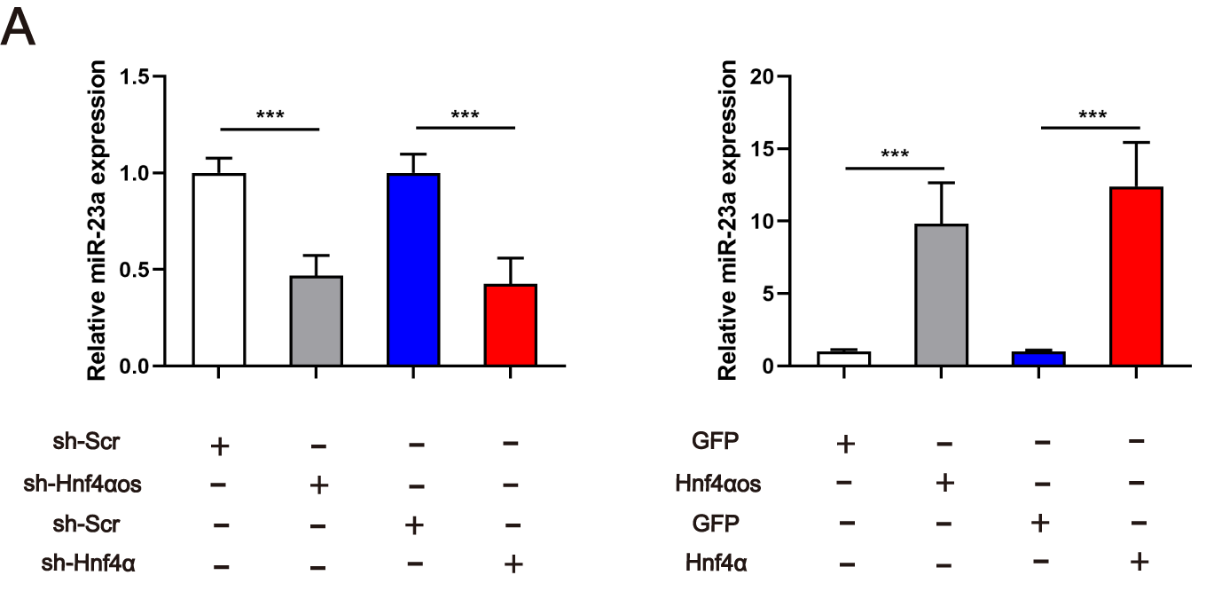


**Fig. S8 Relationship between the expression of miR-23a, *Hnf4αos* and *Hnf4α*. (A)** MiR-23a expression levels after Ad-*Hnf4αos*, Ad-*Hnf4α*, Ad-sh*Hnf4αos*, Ad-sh*Hnf4α* transfection. * P < 0.05, ** P < 0.01, *** P < 0.001.


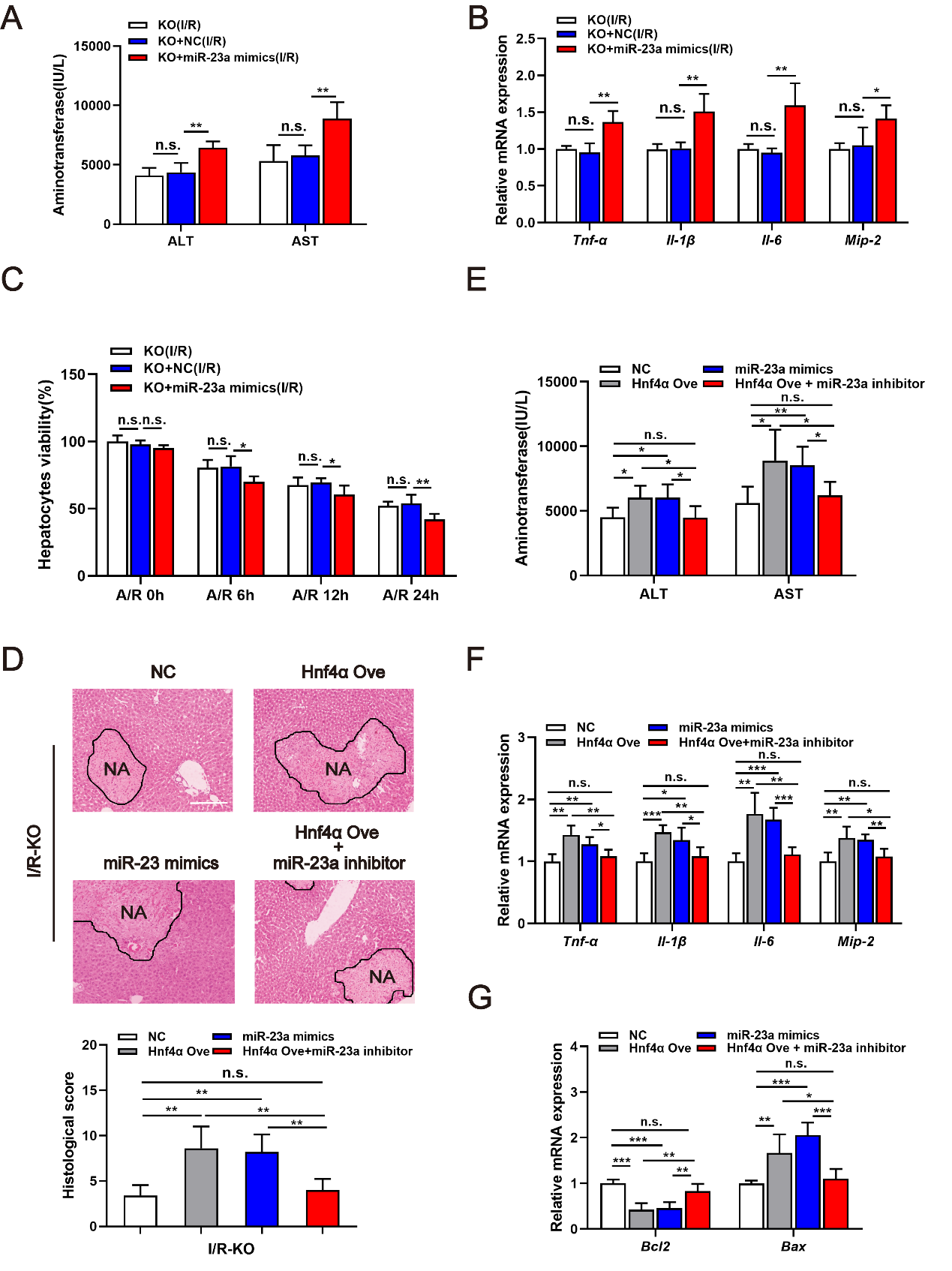


**Fig. S9 MiR-23a exacerbates liver I/R injury. (A)** Serum levels of aminotransferases (ALT and AST) were detected in the Hnf4αos-KO mice subjected to miR-23a mimics after 6h I/R injury. **(B)** The mRNA expression of cytokines/chemokines in in the Hnf4αos-KO mice subjected to miR-23a mimics after I/R injury. **(C)** Hepatocytes viability after A/R treatment by CCK-8 assay. **(D-G)** MiR-23a inhibition attenuated liver damage induced by Hnf4α overexpression. n.s. P > 0.05, * P < 0.05, ** P < 0.01, *** P < 0.001.


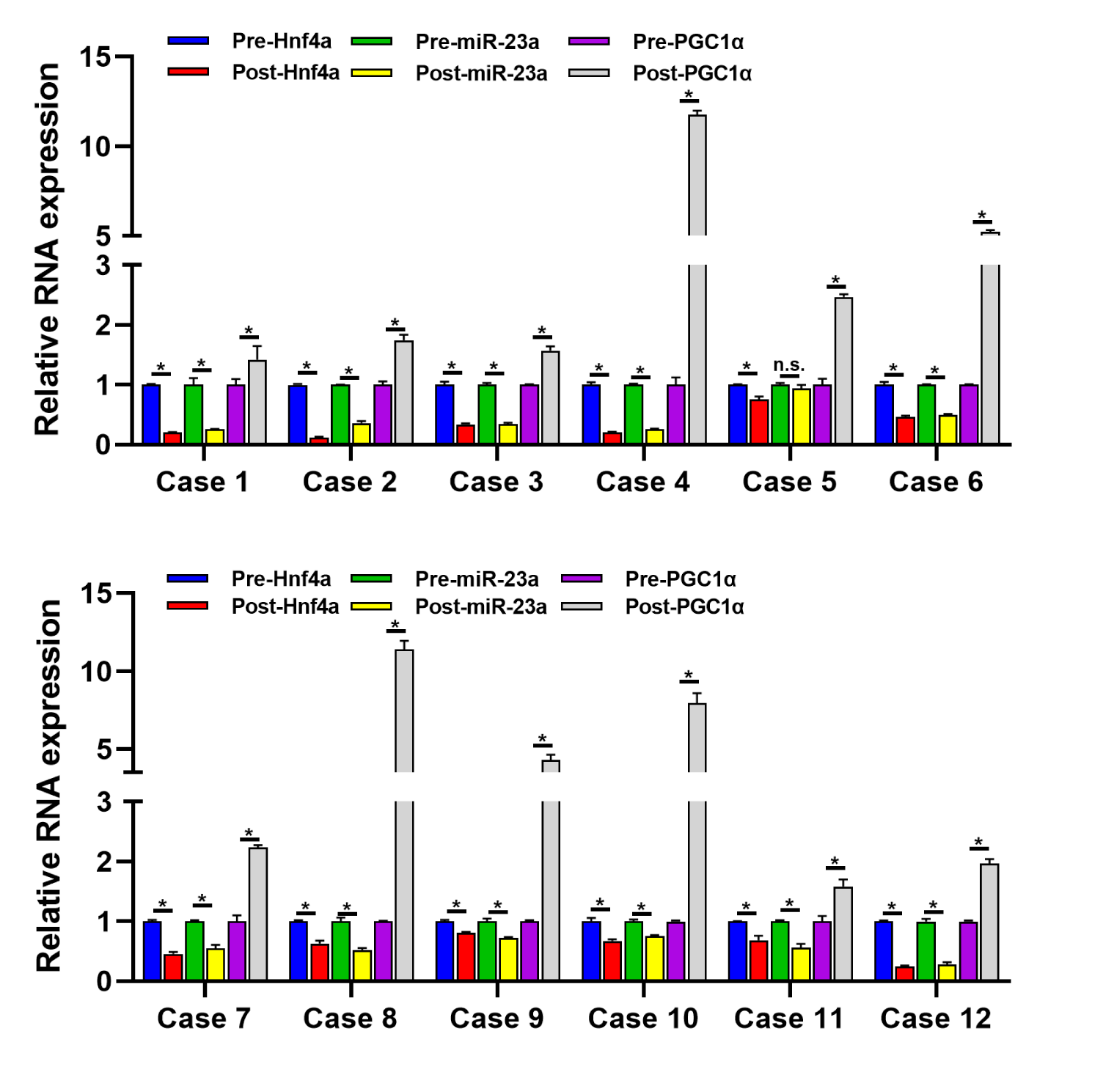


**Fig. S10 *Hnf4α*, miR-23a and *PGC1α* relative expressions were assessed by qRT-PCR in the livers of individuals underwent I/R surgery.** *Hnf4α* and miR-23a expressions were decreased and *PGC1α* expression was increased after I/R surgery. * P < 0.05.
